# The Quaternary Kurobegawa Granite: an example of a deeply dissected resurgent pluton

**DOI:** 10.1038/s41598-021-01562-2

**Published:** 2021-11-11

**Authors:** Hisatoshi Ito, Yoshiko Adachi, Aitor Cambeses, Fernando Bea, Mayuko Fukuyama, Koji Fukuma, Ryuji Yamada, Takashi Kubo, Mami Takehara, Kenji Horie

**Affiliations:** 1 Central Research Institute of Electric Power Industry, Chiba, 270-1194 Japan; 2 Department of Mineralogy and Petrology, University of Granada, 18002 Granada, Spain; 3 Graduate School of Engineering Science, Akita University, Akita, 010-8502 Japan; 4 Department of Environmental System Science, Doshisha University, Kyotanabe, 610-0394 Japan; 5 National Research Institute for Earth Science and Disaster Resilience, Tsukuba, Ibaraki 305-0006 Japan; 6 Asahi Town Board of Education, Toyama, 939-0743 Japan; 7 National Institute of Polar Research, Tokyo, 190-8518 Japan; 8 Department of Polar Sciences, The Graduate University for Advanced Studies, SOKENDAI, Tokyo, 190-8518 Japan

**Keywords:** Volcanology, Environmental impact

## Abstract

The Quaternary Kurobegawa Granite, central Japan, is not only the youngest known granitic pluton exposed on the Earth’s surface, it is one of few localities where both Quaternary volcanics and related plutons are well exposed. Here, we present new zircon U–Pb ages together with whole rock and mineral geochemical data, revealing that the Kurobegawa Granite is a resurgent pluton that was emplaced following the caldera-forming eruption of the Jiigatake Volcanics at 1.55 ± 0.09 Ma. Following the eruption, the remnant magma chamber progressively cooled forming the voluminous Kurobegawa pluton in the upper crust (~ 6 km depth) until ~ 0.7 Ma when resurgence caused rapid uplift and erosion in the region. This is the first study to document the detailed spatiotemporal evolution of resurgent pluton for a Quaternary caldera system. Our new findings may contribute significantly to understanding the fate of active caldera systems that can produce supereruptions.

## Introduction

**Figure 1:**
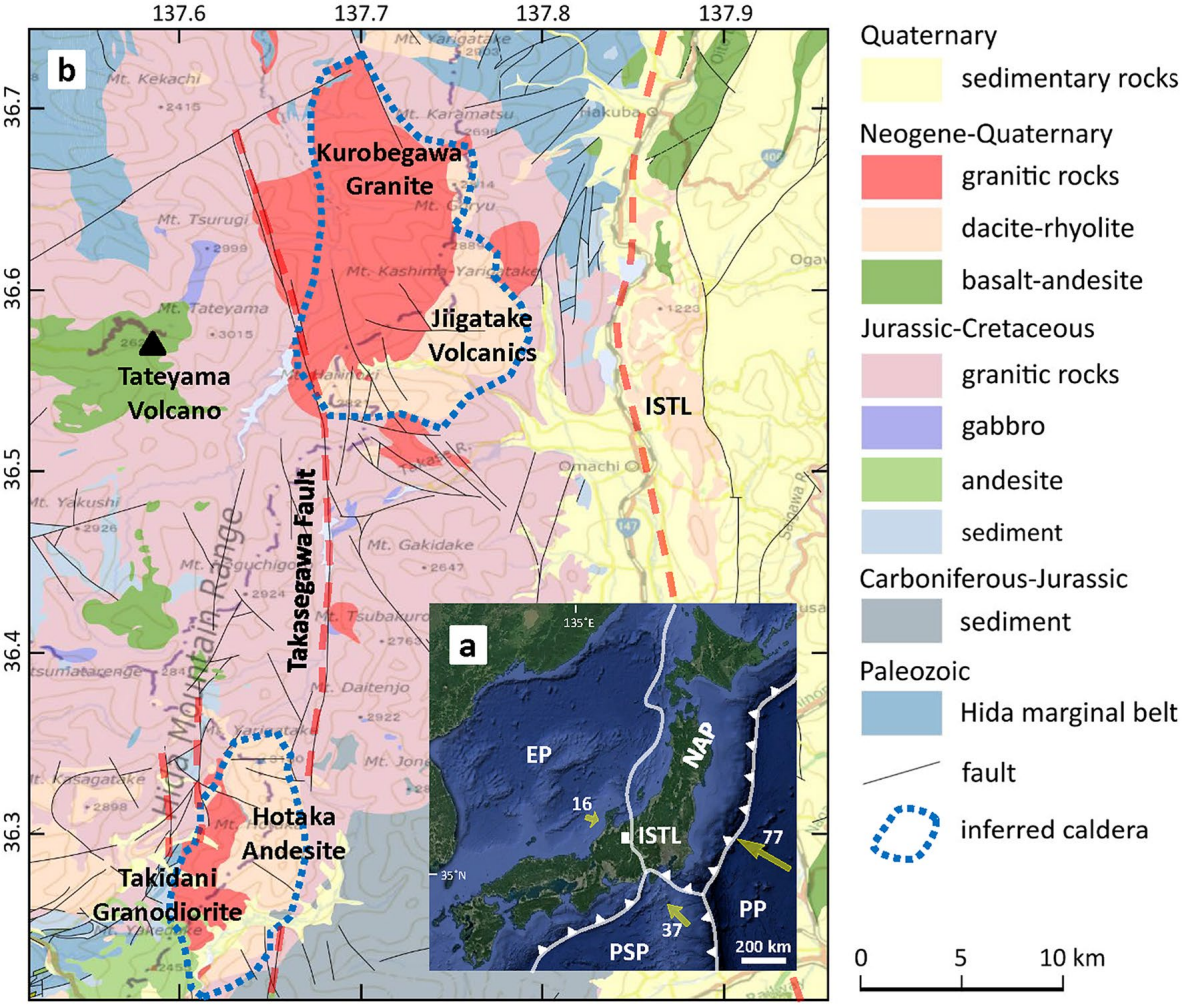
(**a**) Map of Japan with tectonic boundaries (white lines) and the location of the study site shown by a white rectangular. The base map is from Google Earth. (**b**) Geological map of the Hida Mountain Range showing the location of two Quaternary granitic plutons: the Kurobegawa Granite and the Takidani Granodiorite. The plutons lie between N–S tectonic lineaments (red dashed lines). *ISTL* Itoigawa–Shizuoka Tectonic Line, *EP* Eurasian Plate, *NAP* North American Plate, *PSP* Philippine Sea Plate, *PP* Pacific Plate. Yellow arrows in (**a**) indicate plate motion vectors (numbers indicating mm/year) relative to the North American Plate^12^. A black triangle in (**b**) is an active volcano (Tateyama Volcano). Geological map is modified from Seamless digital geological map of Japan 1: 200,000 (Geological Survey of Japan, AIST) using QGIS software (version 3.18.3) (https://qgis.org/ja/site/) and Inkscape (version 1.0.2) (https://inkscape.org/). The base map in (**b**) is the Chiriin Tile (Japan and Its Surroundings) (http://maps.gsi.go.jp) from the Geospatial Information Authority of Japan.

Plutons are an important component of Earth’s lithosphere and it is vital to better understand how they are assembled in the crust. Two different processes have been proposed to explain how plutons are emplaced in the upper crust: (1) plutons represent the remains of large-volume magma chambers that are genetically linked to caldera-forming eruptions^1,2^; or (2) plutons are emplaced incrementally through amalgamation of intrusions over millions of years and consequently not formed by the crystallization of large-volume magma chambers^3–5^. Seismic data may be consistent with protracted timescales of plutonic suite construction because geophysical studies have failed to locate large volumes of melt beneath active volcanic regions^6–10^, although this does not necessarily preclude the existence of voluminous melt^11^. Here, we focus on Earth’s youngest exposed pluton, the Kurobegawa Granite in the Hida Mountain Range (HMR), central Japan (Fig. 1), to better understand pluton assembly.

The Kurobegawa Granite intrudes the Jiigatake Volcanics, which together are inferred to be petrogenetically linked and constitute a volcano–plutonic complex^13^ (Fig. 1b). Harayama et al.^13^ noted that the Kurobegawa Granite may be associated with a resurgent dome^14^ emplaced following the voluminous eruption of the Jiigatake Volcanics, although geochronological evidence to support their idea was lacking. Harayama et al.^13^ and Harayama^15^ also suggested that the rapid uplift and erosion of the Kurobegawa pluton was due to the prevailing E–W compressional stress regime, resulting in the pluton’s near-vertical eastward tilt.

Ito et al.^16^ provided zircon U–Pb age data from 35 localities associated with the Kurobegawa Granite (sensu lato) and revealed that it is the Earth’s youngest exposed pluton amalgamated starting at 10 Ma with multiple episodes of magmatic intrusion; the youngest at ca. 0.8 Ma. In this study, we refer to the Kurobegawa Granite as a Quaternary pluton that intruded after the Jiigatake eruption (sensu stricto). Ito et al.^17^ analyzed modern fluvial (= detrital) zircons collected in the vicinity of the northwestern margin of the Kurobegawa pluton, revealing two magmatic pulses at ~ 2.3 Ma and ~ 0.9 Ma. Spencer et al.^18^ obtained high and low temperature thermochronogical data for the HMR and calculated the highest rates and magnitude of plutonic rock exhumation in Japan—one of the highest worldwide, which they associated with collision of the Izu–Bonin oceanic arc on the Philippine Sea Plate with the Japan arc (Fig. 1a).

Here, we provide new zircon U–Pb ages and geochemical data to better understand the spatiotemporal petrogenetic intrusion history of the Kurobegawa pluton and its predecessor, the Jiigatake Volcanics. We argue that the Kurobegawa Granite is a rare example of a deeply dissected resurgent pluton associated with a Quaternary caldera system and that the resurgence itself played a major role in uplift and erosion.

## Brief description of the Kurobegawa pluton and its associates

The Kurobegawa Granite in the HMR is located to the west of the Itoigawa–Shizuoka Tectonic Line (ISTL), an active plate-bounding fault zone, and ~ 30 km north of another Quaternary sizable pluton, the Takidani Granodiorite (Fig. 1b). Both plutons are also bounded by N–S tectonic lineaments to the west^19^. Moreover, both plutons intrude volcanic rocks produced by caldera-forming eruptions and are interpreted to be the intrusive remnants of the Jiigatake–Kurobegawa and Hotaka–Takidani volcano–plutonic complexes^13,19–21^, although no topographic features of caldera margins remain due to rapid uplift and erosion.

Two stages of mountain building have been suggested for the Quaternary tectonics in the HMR: a period of magmatic intrusion from 2.7 to 1.5 Ma, followed by a period of E–W compressional stress from 1.4 to 0.5 Ma^13,15^. The Takasegawa fault (Fig. 1b), a tectonic lineament > 30 km in length and with a < 200 m wide fracture zone, lies parallel to the ISTL in the middle of the HMR with mylonitized granitic rocks exposed along the lineament^15,22^. It is thought that E–W compressive stress since 1.4 Ma focused along this lineament caused the eastern part of the HMR to be uplifted and tilted eastward, exposing deeper sections of the granite^13,15^; the credibility of this hypothesis will be discussed in this paper.

**Figure 2:**
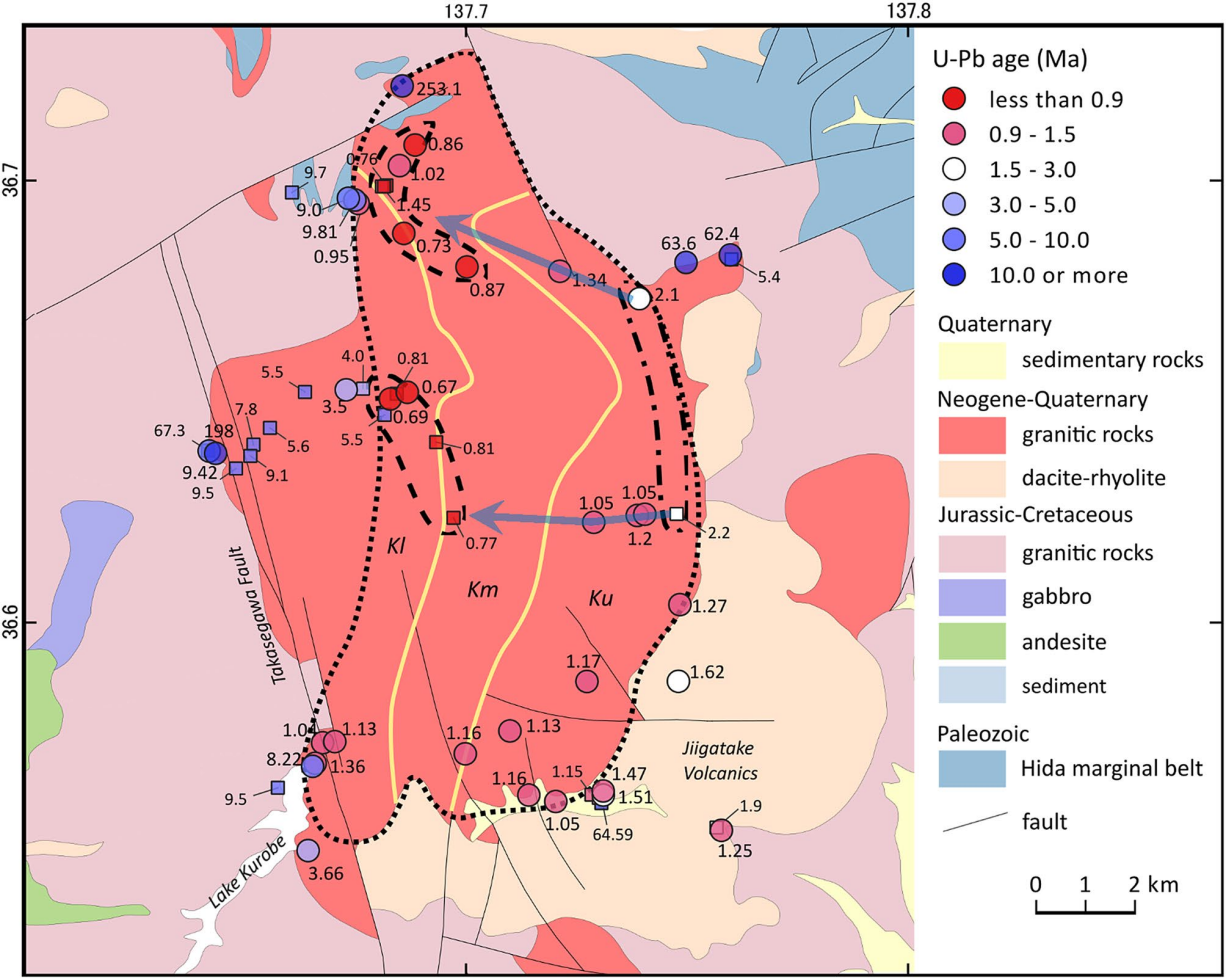
Geological map of the area surrounding the Kurobegawa Granite, showing the sample locations and zircon U–Pb ages. Dotted area denotes the Quaternary Kurobegawa Granite including ~ 2 Ma portion. Large circles and small squares are ages in this study and the literature^16–18^, respectively. Dashed and dash-dot areas are youngest (< 1.0 Ma) and oldest (> 2.0 Ma) portions, respectively. Two blue lines indicate the granite becomes younger to the west. The three lithological units (upper, middle, and lower shown as Ku, Km, and Kl, respectively) of Wada et al.^23^ is delineated by yellow lines. Hida marginal belt comprises ultramafic rocks, sediments and metamorphic rocks. Geological map is modified from Seamless digital geological map of Japan 1: 200,000 (Geological Survey of Japan, AIST) using QGIS software (version 3.18.3) (https://qgis.org/ja/site/) and Inkscape (version 1.0.2) (https://inkscape.org/).

The Kurobegawa Granite is a plutonic complex exposed over an area of ~ 100 km^2^ with vertical exposure from 700 to 2900 m in elevation in the northern HMR. It is divided into three units (upper, middle, and lower) based on its geochemistry and texture^23^ (Fig. 2) and locally intruded the Jiigatake Volcanics at its southeastern border. Along this contact, the granite becomes leucocratic with contact-metamorphism observable in the Jiigatake Volcanics^15,23,24^. The Jiigatake Volcanics are exposed as intra-caldera volcanic deposits of Quaternary age that extend 16 km to the north–south and are locally > 3500 m in thickness^13^. We estimate the volume of Jiigatake Volcanics to be ~ 300 km^3^ of dense rock equivalent (See “Methods” section for the volume estimate). The Jiigatake eruption occurred at least twice^15,25^, forming a composite caldera (the Jiigatake–Shirasawatengu Caldera Complex) coevally.

The Kurobegawa Granite is interpreted to be the remnant of a felsic magma chamber that was zoned as a result of fractional crystallization^23^. It contains abundant microgranular mafic enclaves (MMEs) suggesting that mafic magma was repeatedly intruded into the base of a crystal mush magma body, eventually forming MMEs^23^.

**Figure 3:**
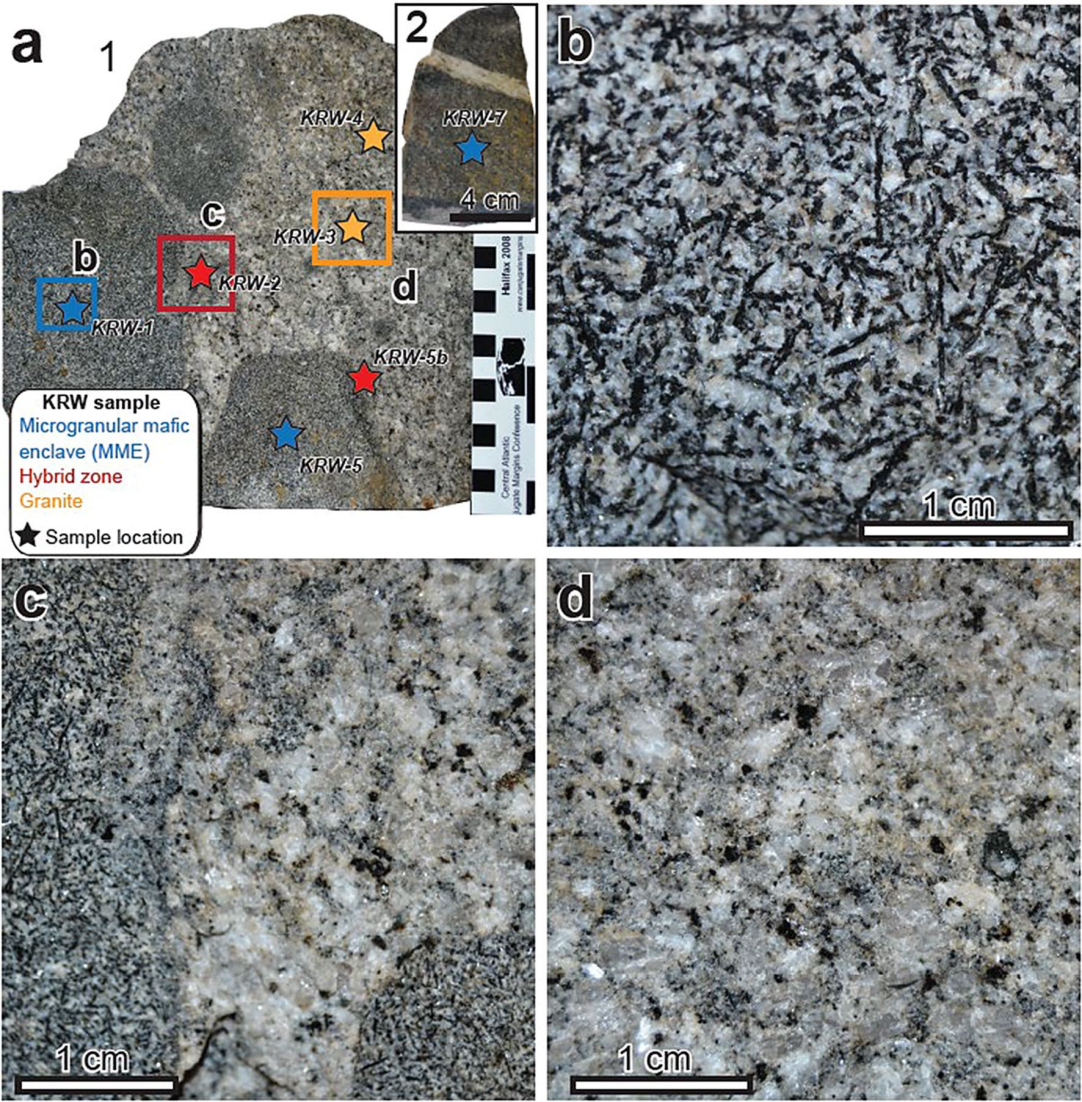
(**a**) Photographs of a sample KRW that includes both felsic and mafic components and an MME (2: inset in **a**) that were analyzed in detail at Granada University. The scale in (**a**) is cm. The sampling site is shown as KRW3 in Supplementary Fig. S1. Photographs (**b**–**d**) are magnified images for subsamples KWR-1, KWR-2, and KWR-3, respectively. Subsamples KWR-4, -5, -5b, and -7 are also shown as stars in (**a**). Photos were taken by Aitor Cambeses.

Sampling was performed to cover all the three units and also to supplement areas that are sparsely dated in the literature^16–18^ (Fig. 2). Zircon U–Pb dating was performed using laser ablation–inductively coupled plasma–mass spectrometry (LA–ICP–MS). Geochemical analyses, including whole rock major and minor element analyses and trace element analyses in zircon, were performed to better understand magmatic history of the Jiigatake–Kurobegawa volcano–plutonic complex. Detailed investigation of a sample that includes both felsic and mafic components and an MME sample (samples name: KRW, Fig. 3) was also performed using sensitive high-resolution ion microprobe (SHRIMP) for U–Pb dating and various geochemical techniques.

## Results

### Zircon U–Pb analyses

LA-ICP-MS zircon U–Pb ages from 35 sites were newly obtained in this study (Fig. 2; Supplementary Table S2). Representative cathodoluminescence (CL) images with U–Pb ages are shown in Supplementary Fig. S2. The CL images generally show oscillatory and patchy zoning.

Four samples from the Jiigatake Volcanics were dated, yielding 1.62 ± 0.13 Ma (KRB29; see Supplementary Fig. S1 for location), 1.51 ± 0.23 Ma (KRB31), 1.25 ± 0.70 Ma (KRB37), and 1.47 ± 0.18 Ma (KRG40). These four ages overlap within uncertainty and yield a weighted mean age of 1.55 ± 0.09 Ma (error: 95% confidence; MSWD = 0.93). Notably, the Jiigatake Volcanics contain some elongated zircons rich in melt inclusions (e.g., KRG40-9).

The granitic rocks we analyzed ranged from ~ 250 to 0.7 Ma. Assuming that the age of the Kurobegawa Granite is younger than the age of the Jiigatake Volcanics (1.55 ± 0.09 Ma), we identify 20 of the 30 granite samples to be associated with the Kurobegawa Granite. Older samples include the following: A granite (KRB20) at the northeastern margin of the Kurobegawa Granite yielded 2.10 ± 0.16 Ma. From the western margin of the Kurobegawa Granite, two granite samples yielded 3.50 ± 0.14 Ma (KRG12) and 3.66 ± 0.18 Ma (YH126), four granite samples (KRB08; KRB23; KRG14; KRG24) yielded 10–8 Ma, and a metamorphosed granite (KRG16) yielded 198.0 ± 3.5 Ma. Along the northeastern margin of the Kurobegawa Granite, a granite (KRB19) and a rhyolitic welled tuff (KRB25) yielded 63.6 ± 1.3 Ma and 62.4 ± 2.20 Ma, respectively. Finally, a granite from the northern margin of the Kurobegawa Granite (KRB11) yielded 253.1 ± 9.2 Ma.

For the Kurobegawa Granite, a sample collected in the northern part of the pluton (KRB18) containing both the felsic and mafic components were separately dated, which yielded 0.91 ± 0.20 Ma and 1.06 ± 0.12 Ma, respectively. Because the ages overlap within error and both dates were obtained from the same sample, they are shown as one point and a weighted mean age of 1.02 Ma on the age distribution map (Fig. 2).

As a test of the accuracy of our LA–ICP–MS ages, samples KRB04 and KRB08 were collected from the sites of JP08 and JP16 of Spencer et al.^18^, respectively. New LA–ICP–MS zircon U–Pb ages for KRB04 and KRB08 are 1.05 ± 0.07 Ma and 9.00 ± 0.12 Ma, respectively. Previously reported zircon U–Pb ages for JP08 and JP16 are 1.14 ± 0.14 Ma and 9.11 ± 0.29 Ma, respectively, measured using a different LA–ICP–MS system at Curtin University, Australia^18^. The consistency of these ages supports the robustness of the U–Pb ages in this study.

A granite portion (KRW-3) of the KRW sample (Fig. 3) from the northern part of the pluton was dated by both LA–ICP–MS and SHRIMP methods, yielding 0.73 ± 0.03 Ma (Supplementary Table S2) and 0.77 ± 0.04 Ma (Supplementary Table S3), respectively. Representative CL images and SHRIMP U–Pb ages are shown in Supplementary Fig. S3.

### Thermobarometric results

Zircon saturation temperatures for the Kurobegawa Granite were calculated following Watson and Harrison^26^ (Supplementary Fig. S4a), yielding a range from 700 to 850 °C. Zircon saturation temperatures for the Jiigatake Volcanics, a 2 Ma granite, and a 10 Ma granite were also plotted together with the Kurobegawa Granite (Supplementary Fig. S4b). The calculated temperatures of the Jiigatake Volcanics are generally warmer than the Kurobegawa Granite.

**Figure 4:**
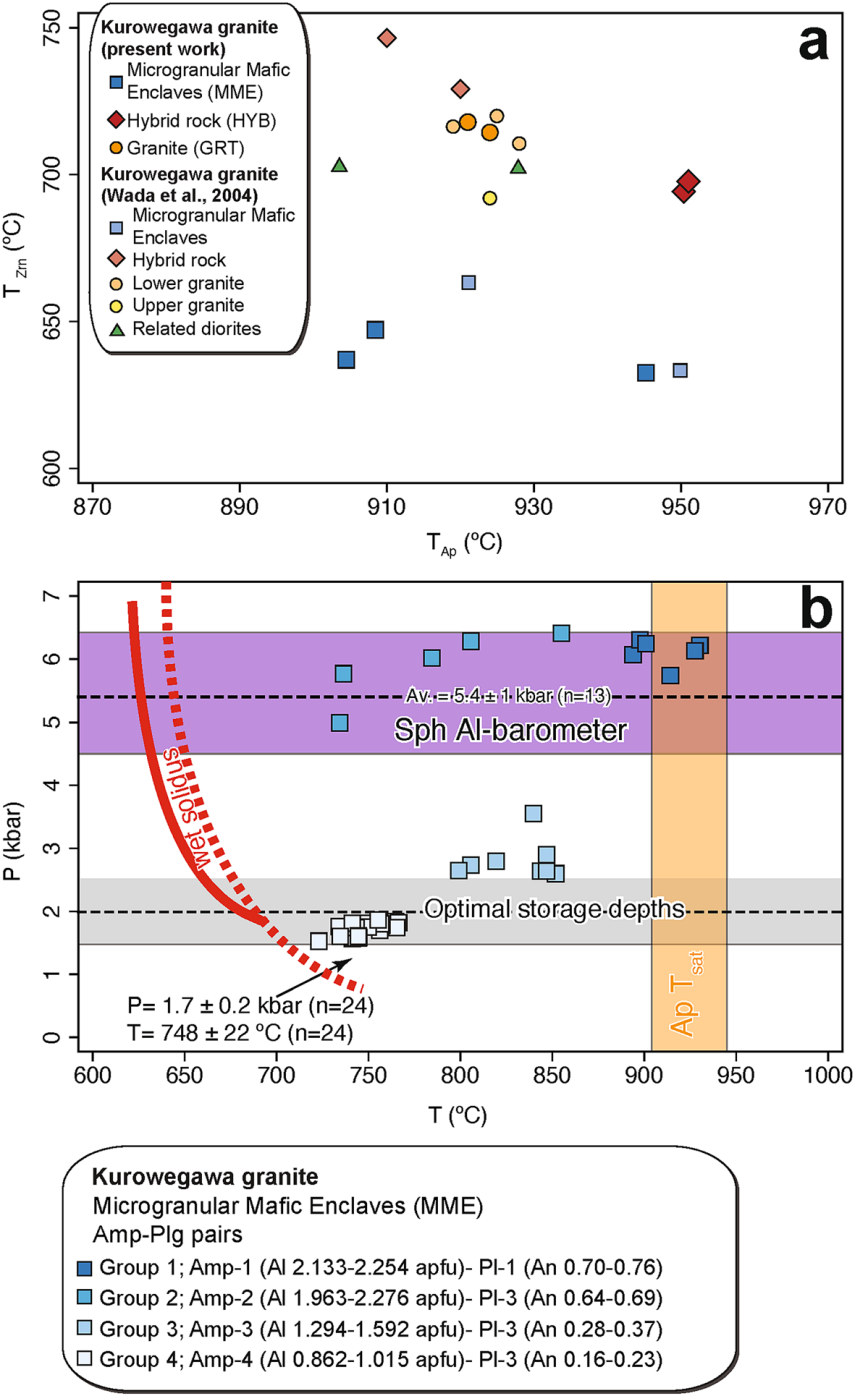
Temperature and pressure estimates for the Kurobegawa Granite. (**a**) Calculated zircon saturation temperature (T_Zrn_) vs apatite saturation temperature (T_Ap_) using the KRW sample (Fig. 3) and data from Wada et al.^23^ (**b**) Pressure vs temperature for various amphibole (Amp)-plagioclase (Pl) pairs in MMEs. We grouped four Amp-Pl pairs based on total Al content (atoms per formula unit: apfu) in Amp and anorthite (An) content in Pl. Temperatures were calculated using the Amp-Pl thermometer of Molina et al.^27^ for groups 1 and 2 and Holland and Blundy^28^ for groups 3 and 4. Pressures were estimated using the Al in amphibole barometer^29^. The pressure range by the Al in sphene (Sph) barometry^30^ and the temperature range by apatite (Ap) saturation thermometry are also plotted. The red dotted line shows the experimentally determined water-saturated solidus for tonalite^31^ and the red solid line is the water-saturated solidus calculated using rhyolite-MELTS for diorite^32^. The transparent-grey area represents the proposed optimal emplacement window of shallow magma chambers (P = 2 ± 0.5 kbar), estimated using thermo-mechanical models^33^.

Zircon and apatite saturation temperatures were also calculated using the KRW sample and Wada et al.^23^ data (Fig. 4a). In general, the apatite saturation temperatures are 150–200 °C higher than the zircon saturation temperatures, which is in agreement with the observation that apatites are an early crystallizing phase and occur as inclusions in zircon.

**Figure 5:**
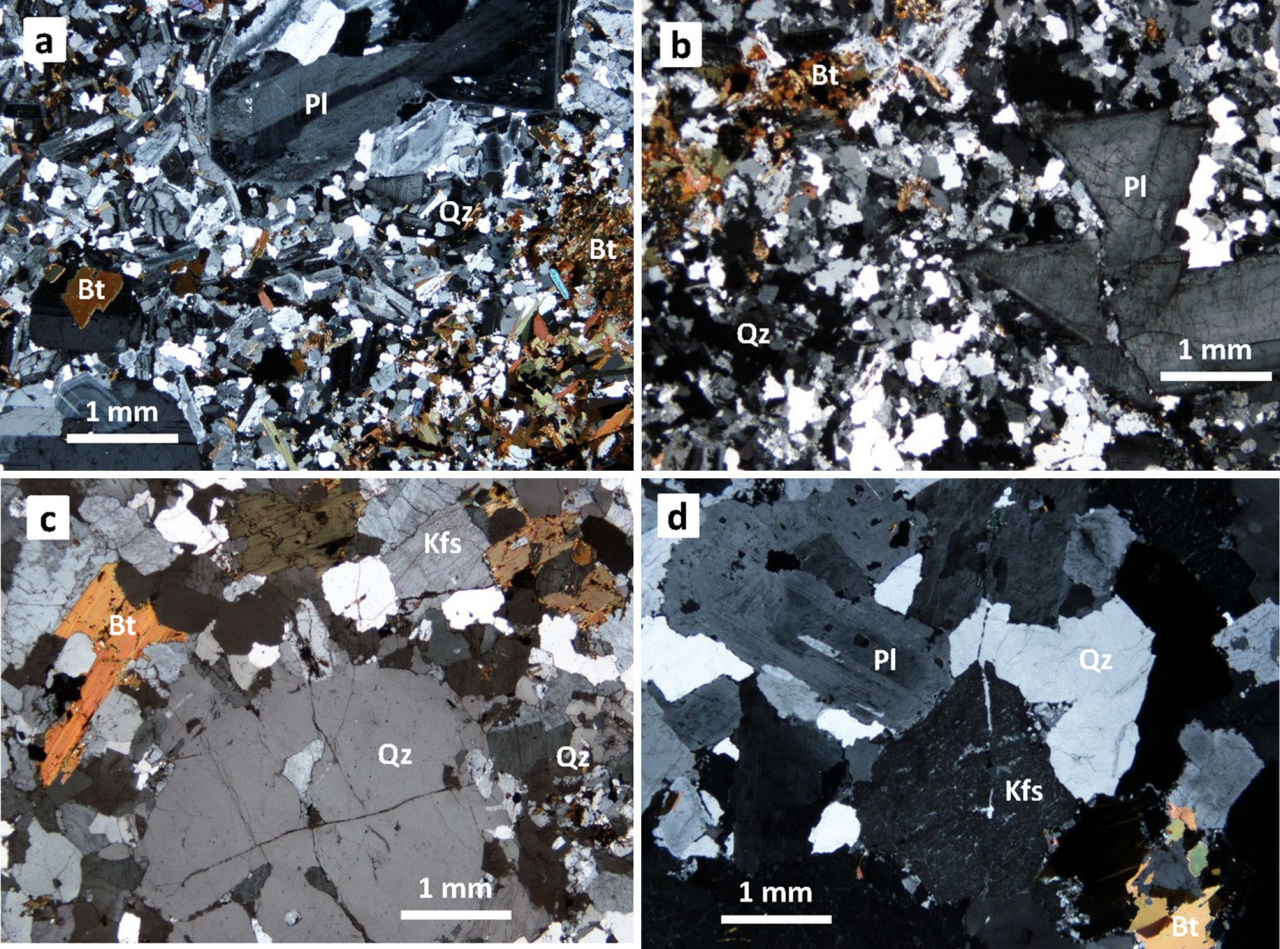
Photomicrographs of the Kurobegawa Granite (**a**–**c**) and a ~ 10 Ma granite (**d**) taken in cross-polarized light. (**a**) Typical porphyritic textures with micro crystals of quartz, plagioclase and biotite at the northwestern margin of the granite at Loc. KRB16. (**b**) Groundmass quartz shows a strong dynamic recrystallization and fracturing of a large plagioclase phenocryst near the Jiigatake Volcanics at Loc. KRB30. Many xenocrystic zircons were identified in this sample. (**c**) Groundmass quartz shows a weak dynamic recrystallization, and a quartz phenocryst shows dissolution at the south of the granite at Loc. KRB27. (**d**) Equigranular granite showing indications of neither tectonic shear nor fluid migration located to the west of the northern Kurobegawa Granite at Loc. KRB23. *Qz* quartz, *Pl* plagioclase, *Bt* biotite, *Kfs* potassium feldspar.

MMEs in the KRW sample were further analyzed by amphibole-plagioclase thermobarometry to evaluate the depth of emplacement of the KRW. The magmatic temperature and pressure of the MMEs were estimated at 748 ± 22 °C and 1.7 ± 0.2 kbar, respectively (Fig. 4b; see “Supplementary Information” for further explanation). The relatively low emplacement pressure (or shallow depth) of 1.7 kbar is in accordance with the fact that the Kurobegawa Granite is mostly porphyritic (Fig. 5a–c) with miarolitic cavities extensively developed throughout the pluton^23^. The depth estimate is within the optimal emplacement window for shallow magma chambers, estimated using thermo-mechanical models^33^ (Fig. 4b).

### Petrography and geochemical results

We studied in detail a Kurowegawa Granite sample (KRW) that includes both felsic and MME components (KRW-1, -2; -3, -4, 5 and 5b) and an MME sample (KRW-7) (Fig. 3). We processed the sample to obtain representative portions of MME (KRW-1, -5 and -7), host granite (KRW-3 and -4) and intermingled zone (KRW-2 and -5b) in order to better quantify the textures and geochemical compositions. Whole rock major and trace element analytical results for these samples are shown in Supplementary Table S4.

The MMEs are tonalites characterized by a medium-grained, equigranular hypidiomorphic texture. They contain plagioclase, amphibole, biotite and interstitial alkali feldspar and quartz. Accessory phases include apatite, sphene, epidote, zircon, magnetite and pyrite. The texture of the host granite is coarse- to medium-grained and inequigranular. It is composed of large crystals of plagioclase, alkali feldspar, biotite and quartz in a fine-grained matrix with the same mineralogy and minor amphibole. Accessory phases in the granites are apatite, zircon, monazite, epidote, ilmenite and magnetite.

In the intermingled zone, there are recognizable phases from both MMEs and host granites. However, in these areas, main phases show disequilibrium textures, including: normal and reversed oscillatory-zoned plagioclase, spongy-cellular plagioclase included in large plagioclase, patchy-zoned amphibole, and ocelar quartz megacrysts showing a corona of mafic minerals overgrowth with small grain quartz. Textures and mineral chemistry allow identification of different types of phases that formed during different crystallization stages.

Chondrite normalized rare earth element (REE) patterns for whole rock and zircon from the Kurobegawa Granite, the Jiigatake Volcanics, and older granites are shown in Supplementary Fig. S5 and Supplementary Fig. S6, respectively. Supplementary Fig. S5a shows that the Jiigatake Volcanics (dotted lines) are enriched in REE relative to the Kurobegawa Granite, with the exception of KRB30. Sample KRB30, collected near the Jiigatake Volcanics, is the most REE enriched, indicating that the magma from which the KRB30 zircon crystallized at ~ 1.3 Ma was most evolved. This sample also contains a high proportion of xenocrystic (> 50 Ma) and antecrystic (> 2 Ma) zircons (Supplementary Fig. S2; Supplementary Table S2), which may indicate that this magma assimilated older intrusive material in the shallow upper crust.

See Supplementary Fig. S7 for a summary of zircon Hf isotope compositions for the Kurobegawa Granite compared to the measured U–Pb ages. The initial ^176^Hf/^177^Hf ratios, expressed as εHf(t), predominantly range from − 4 to − 1, which are comparable with the results from Spencer et al.^18^. Spencer et al.^18^ also measured older (~ 65 Ma and ~ 10 Ma) granites from surrounding areas. The overall agreement with the Kurobegawa Granite compositions and the older granites indicates that the Kurobegawa magma assimilated similar upper crustal material. The εHf(t) value for the mafic part (KRB18M) ranged from − 3.4 to − 0.9, which is more tightly clustered than the felsic part (KRB18F) ranging from − 2.9 to + 3.2, suggesting that the MME derives from a more homogenies (and deeper) source than the felsic part of the granite. Larger variation of εHf(t) in zircon from the felsic part shows the variable degree of assimilation during the formation of the Kurobegawa pluton.

## Discussion

Although the newly obtained U–Pb ages generally agree with those in the literature^16–18^, we can now more accurately constrain the history of the Jiigatake–Kurobegawa volcano–plutonic complex.

Based on our new results, the U–Pb age of the Jiigatake Volcanics is more tightly constrained at 1.55 ± 0.09 Ma. Previously, the published ages for this formation were 1.55 ± 0.28 Ma (K–Ar groundmass)^34^ and 1.90 ± 0.50 Ma (U–Pb zircon)^16^. The accordance of our new U–Pb zircon with previously published K–Ar groundmass ages confirms that the Jiigatake magma accumulated and erupted at 1.55 ± 0.09 Ma.

The geochronology for the remnant magma chamber that, upon cooling, eventually became Kurobegawa Granite was not so simple. Based on our new and previous ages, magmatic activity along the north-eastern margin of the pluton (dash-dot lines in Fig. 2) started between 2.2 and 2.1 Ma; ~ 0.6 million years earlier than the Jiigatake volcanic activity. Following the eruption of the Jiigatake Volcanics at 1.55 Ma, Kurobegawa magmatism migrated westward, defined by ages for the Kurobegawa Granite that become younger toward the west (blue arrows in Fig. 2). Kurobegawa magmatism continued until 0.8–0.7 Ma along the north-western margin (dashed lines in Fig. 2). The youngest ages correspond to the areas of modern high temperature (> 60 °C) hot springs unrelated to volcanic activity^34^, suggesting remnant heat from the cooling Kurobegawa Granite is likely driving hydrothermal activity. In contrast, the southern part of the Kurobegawa Granite yields ages mostly 1.4–1.0 Ma, indicating that magma chamber was replenished soon after the voluminous 1.55 Ma Jiigatake eruption and until ~ 1.0 Ma.

**Figure 6:**
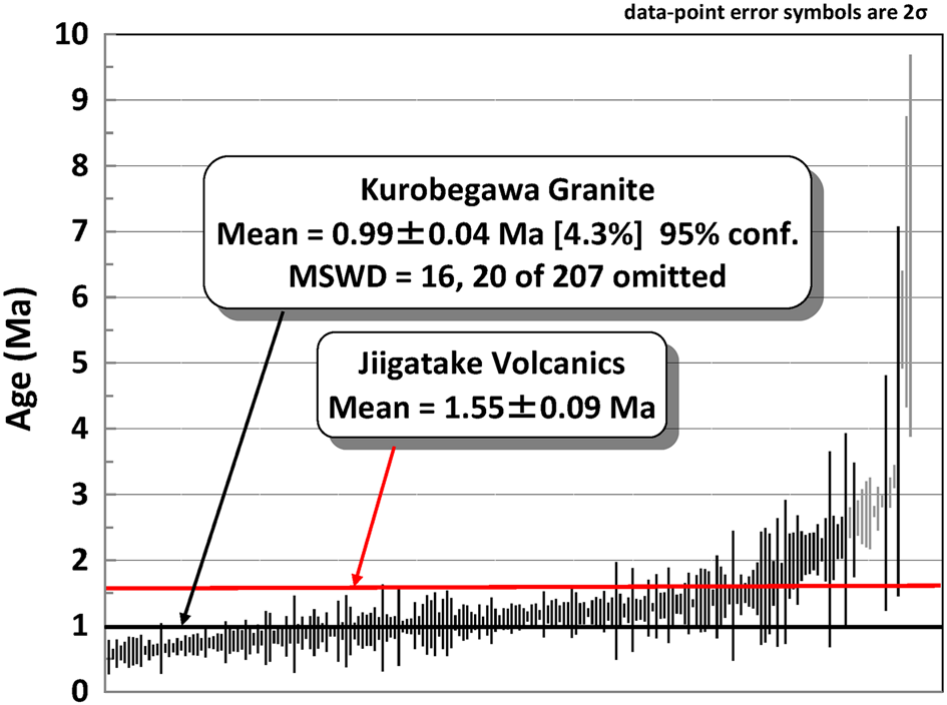
^238^U–^206^Pb age distributions for the Kurobegawa Granite (including ~ 2 Ma portion) zircons younger than 10 Ma (7 grains > 10 Ma are omitted). Individual grain ages with 2σ uncertainty are arranged in rank order. Analyses in grey represent statistical outliers and were excluded. Black and red horizontal bars represent mean U–Pb age of the Kurobegawa Granite and the ~ 1.55 Ma Jiigatake eruption, respectively. *MSWD* mean square weighted deviation.

**Figure 7:**
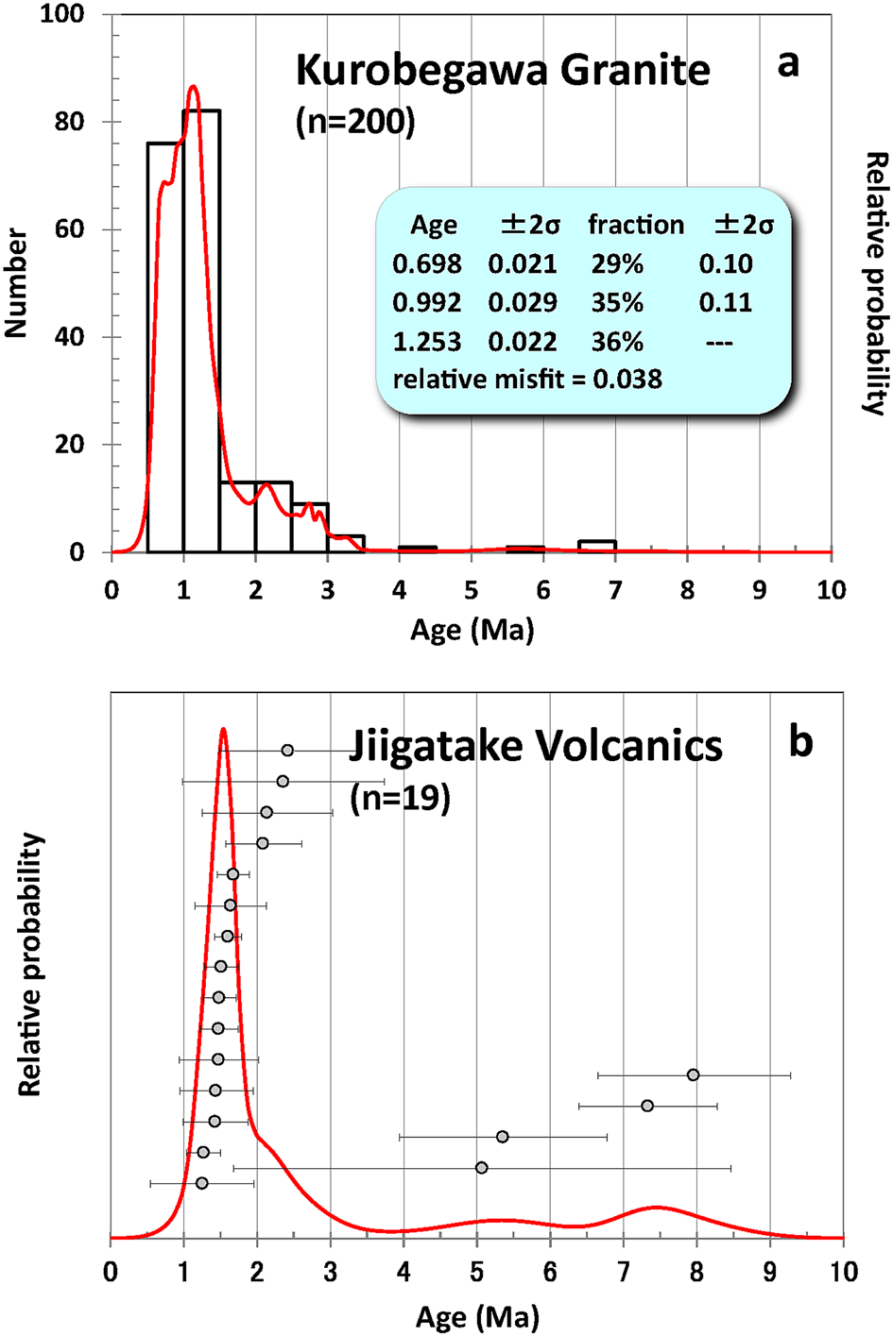
^238^U–^206^Pb age distributions (histogram, probability density plot) for zircon younger than 10 Ma for (**a**) the Kurobegawa Granite (including ~ 2 Ma portion) and (**b**) the Jiigatake Volcanics. The inset in (**a**) shows the result of age peak deconvolution using Isoplot^35^. The deconvolution was performed for ages < 1.55 Ma to analyze grouping of zircon ages after the ~ 1.55 Ma Jiigatake eruption. In (**b**), individual grain ages with 2σ uncertainty are also plotted. n = number of grains plotted.

The weighted mean of all the ages from the Kurobegawa pluton (including ~ 2 Ma portion) yields 0.99 ± 0.04 Ma (95% confidence level; MSWD = 16; n = 187) (Fig. 6). Therefore, zircon forming magmatic activity may have culminated 0.5–0.6 million years after the Jiigatake eruption in a broad sense. However, the relatively high MSWD of 16 for this age suggests it is not a single population. The probability density plot of zircon crystallization ages for the Kurobegawa pluton ages (Fig. 7a) has multiple age peaks, compared to the well-defined single age peak for the Jiigatake Volcanics (Fig. 7b). We have deconvoluted the peak spectra for the Kurobegawa pluton ages using the Unmix Ages program^35^ assuming three components. If we take the grouping of zircon crystallization ages to represent timing of magma intrusion and/or reheating of pre-existing mush, the result shows that the Kurobegawa pluton experienced three magmatic pulses at 1.25 ± 0.02 Ma (fraction: 36%), 0.99 ± 0.03 Ma (35%), and 0.70 ± 0.02 Ma (29%) (Fig. 7a). A similar result is also obtained using IsoplotR’s^36^ radial plot (Supplementary Fig. S8) confirming these populations of ages are robust. Although it is possible that the Kurobegawa pluton experienced continuous, prolonged intrusion of magma from ca. 1.4 to 0.7 Ma, the large number of zircon analyses presented here allow us to resolve three statistically distinct pulses that we interpret as magma intrusion events.

Harayama et al.^13^ assumed nearly-vertical eastward tilting of the entire Jiigatake–Kurobegawa volcano–plutonic complex due to E–W compressional stress regime. One main reason is that mylonitic textures are observed in the western margin of the Kurobegawa Granite. However, this is unlikely because at the northwestern margin of the Kurobegawa Granite, no mylonitic texture has been identified in the Kurobegawa Granite (KRB16), nor in the 10 Ma granite to the west (KRB23) (Fig. 5a,d; Supplementary Fig. S1). In this area, mylonite exists to the west of the 10 Ma granite. Therefore, we assume that the N–S mylonite zone formed before 10 Ma and that both the 10 Ma granite and the Kurobegawa Granite intruded along the mylonite zone. Harayama et al.^13^ identified mylonitic texture in the western margin of the Kurobegawa Granite at different localities from ours, but based on their descriptions we assume they may have misidentified the Kurobegawa Granite.

### Resurgence of the Kurobegawa granite

As noted above, it is unlikely that the Kurobegawa Granite tilted almost vertically to the east by the regional E–W compression^13,15^. Similarly, the suggestion that upper units lie progressively to the east^23^ (Fig. 2) is doubtful because this assertion is based on the hypothesis of eastward tilting. It is also unlikely that the entire Kurobegawa Granite experienced a melting phase as a whole as envisaged by Wada et al.^23^ and a large chamber model may not be applicable here based on our new geochronology interpretation. Instead, the Kurobegawa Granite magma likely accumulated and progressively filled the void space caused by the 1.55 Ma Jiigatake eruption from the south and east to the north and west, in a broad sense (Fig. 2). Intrusion of this magma resulted in resurgence, which caused rapid uplift and erosion. Hence, we propose the Kurobegawa Granite is the result of resurgent magmatism because it is spatially restricted to the presumed caldera area (Fig. 1b), the location of which suggested by previous authors^13,15,19^. Additionally, magmatism occurred at shallow crustal levels immediately after the voluminous (~ 300 km^3^) Jiigatake eruption. Assuming that the outcropping Kurobegawa Granite magma solidified at ~ 6 km depth (equivalent to 1.7 kbar assuming a mean crustal density of 2.75 g cm^−3^) at ~ 1 Ma, the uplift/erosion rate is ~ 6 mm/year. Here, we interpret magmatic resurgence to be the dominant driving force for the observed rapid uplift^37^. This is similar, for example, to Toba Caldera, Indonesia, where resurgence caused parts of the Samosir Island to be uplifted 700 m over the past 33,000 year, equivalent to 21 mm/year^38^.

Recently, the intrusion depth and uplift/erosion rate of a ~ 5 Ma granite to the west of the Kurobegawa Granite were estimated ~ 6–11 km and 0.93–2.5 mm/year, respectively^25^. Therefore, the uplift/erosion rate of the Kurobegawa pluton was higher than the surrounding areas, which also supports the resurgent uplift of the pluton.

Prior to this study, identification of magmas driving resurgence in Quaternary systems could only be imaged by geophysical tools such as seismicity, gravity, and resistivity^39,40^. The Kurobegawa Granite is a rare case globally where the deep interior of a Quaternary caldera system can be investigated in the field. On the contrary, the rapid uplift is not restricted to the Kurobegawa Granite exposure, but it extends to the whole HMR^18^. Therefore, we suspect a deeper and more voluminous magmatic reservoir exists beneath the HMR (i.e., larger than the areal extent of the Kurobegawa Granite exposure) as in the case for other large caldera systems such as Toba^41^. Matsubara et al.^6^ found two low seismic velocity zones at depths of 2–4 km and 12–20 km beneath Tateyama Volcano, an active volcano to the west of the Kurobegawa Granite (Fig. 1b) and assumed they correspond to partially molten rocks or magmatic reservoirs. We assume deep magmatic reservoirs such as the 12–20 km magmatic reservoir imaged by Matsubara et al.^6^ likely contribute to the regional uplift of the HMR as a whole. This deep reservoir likely feeds hotter, mafic magma into the base of the shallower Kurobegawa reservoir, similar to models envisaged by other researchers^42,43^.

### Tectonic and magmatic evolution in the Hida Mountain Range (HMR)

**Figure 8:**
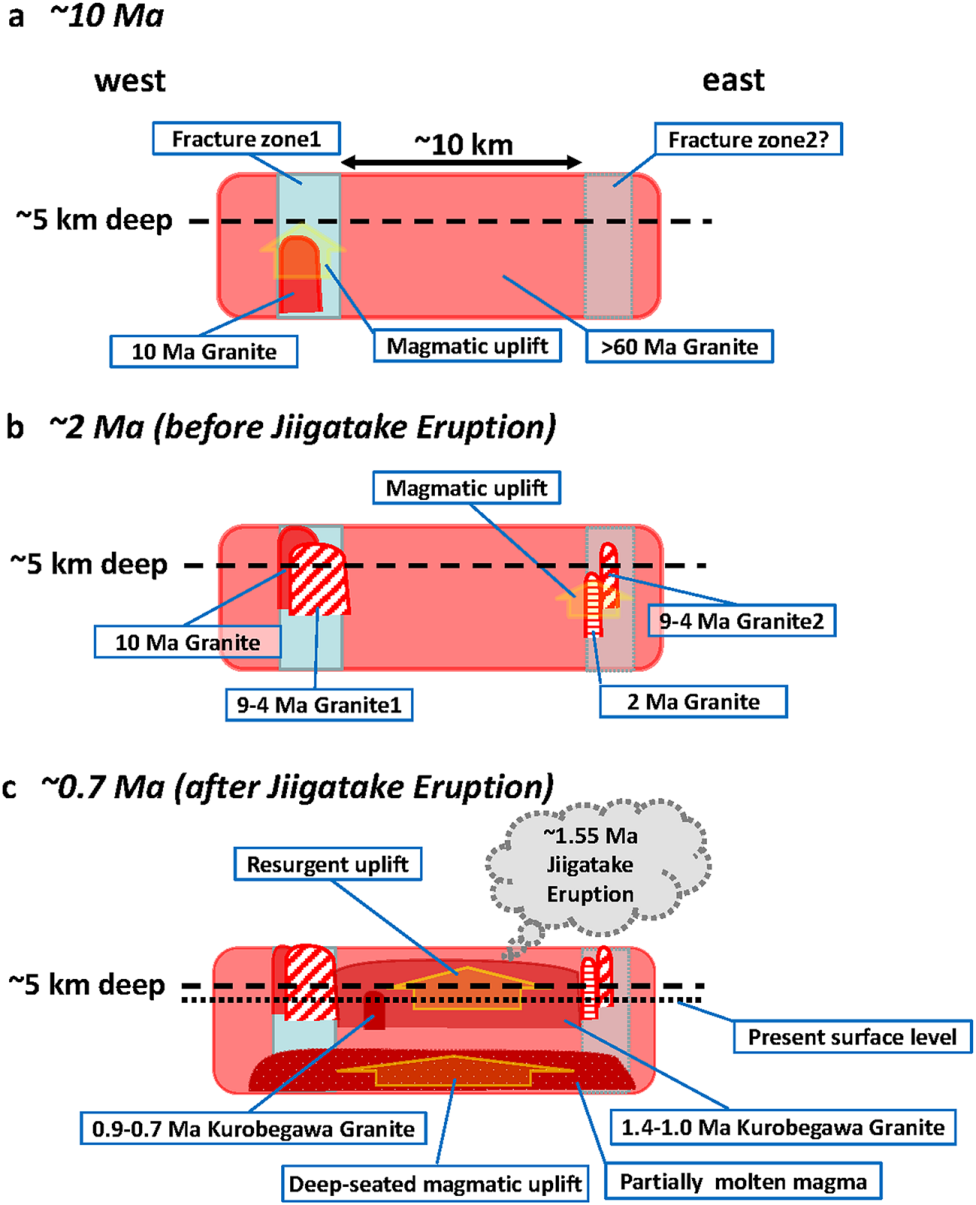
Simplified model of tectonic and magmatic evolution in the Hida Mountain Range at ~ 10 Ma (**a**), ~ 2 Ma (**b**), and ~ 0.7 Ma (**c**). The image was drawn by Hisatoshi Ito.

We present a model for the tectonic and magmatic evolution in the HMR as schematic snapshots at ~ 10 Ma, ~ 2 Ma, and ~ 0.7 Ma (Fig. 8). In the HMR, especially around the Kurobegawa Granite area, granite-producing magmatism prevailed at ~ 65 Ma^16^ (Fig. 8a). Ito et al.^16^ assumed that the Kurobegawa Granite intruded ~ 65 Ma granite because of the existence of ~ 65 Ma xenocrystic zircons in the Kurobegawa pluton. This is further corroborated by the existence of ~ 60 Ma xenocrysts at the southeastern part of the pluton (KRB30; Supplementary Fig. S2). N–S trending fracture zones, such as ISTL and the Takasegawa Fault (Fig. 1), developed as a result of (1) back-arc spreading (Japan Sea opening) which culminated at ~ 15 Ma^44^ and/or (2) collision of the Izu-Bonin oceanic arc with the Japan arc since ~ 13 Ma at the latest^45^.

At ~ 10 Ma (Fig. 8a), granites intruded along N–S trending faults especially along the Takasegawa Fault and its branch faults (Fig. 2). Granite intrusion occurred episodically to ~ 2 Ma^16^. This magmatism associated with the Takasegawa Fault occurred mainly to the west of the presently exposed Kurobegawa Granite area, whereas less voluminous magmatism also occurred to the east of the Kurobegawa Granite (Fig. 8b) based on the identification of ~ 5 Ma and ~ 2 Ma granites near the northeastern margin of the Kurobegawa Granite (Fig. 2). We have also analyzed 9–4 Ma xenocrystic zircons in samples from the eastern margin of the pluton and adjacent Jiigatake Volcanics (e.g. samples KRB29, KRB30 in Supplementary Table S2). Therefore, we assume the existence of another fracture zone (Fracture zone2 in Fig. 8a) to the east of the Kurobegawa Granite.

At 1.55 ± 0.09 Ma, a caldera-forming Jiigatake eruption occurred where the Jiigatake–Kurobegawa complex is presently exposed. Subsequently, resurgence occurred until ~ 0.7 Ma accompanied by rapid uplift and erosion together with more regional magmatism beneath the resurgent pluton. This history has resulted in the present-day exposure of the Kurobegawa Granite (Fig. 8c).

Regionally, the HMR experienced three voluminous eruptions in the Quaternary: two eruptions at ca. 1.76 Ma and 1.75 Ma related to the Hotaka Andesite^46^ (Fig. 1b) in addition to the ~ 1.55 Ma eruption producing the Jiigatake Volcanics (this study). Tectonically, the subduction direction of the Philippine Sea Plate (Fig. 1a) changed from NNW to NW at 3 Ma^47^, which likely caused drastic stress change along the N–S lineaments in the HMR from transpression (?) to compression. This may explain why these relatively large eruptions occurred at 1.76–1.55 Ma and why no voluminous (> 200 km^3^) eruptions happened since in the HMR.

### Implication of pluton formation

Based on new zircon crystallization ages, the Kurobegawa pluton formed mostly ~ 0.5–0.6 million years after the ~ 1.55 Ma caldera-forming Jiigatake eruption. Therefore, in a broad sense, it represents the remain of a large-volume magma chamber^1,2^. However, the pluton was not formed by a single (monotonic) cooling of a magma chamber, but it cooled progressively to the west in the northern half of the pluton. This may indicate the resurgent magma remained hot or was reactivated in the northwestern part of the pluton. The Kurobegawa pluton also contains a ~ 2 Ma component along its eastern margin that was emplaced prior to the Jiigatake eruption. In this sense, the pluton was formed incrementally through amalgamation of intrusions^3–5^ in a timescale of hundreds of thousands of years. Therefore, the Kurobegawa pluton may represent a hybrid type of the two pluton formation mechanisms. Additionally, the ~ 10–4 Ma plutons west of the Kurobegwa pluton (Fig. 2) formed incrementally over millions of years^16^, with no evidence of caldera formation at that time.

## Conclusions

Earth’s youngest exposed granite, the Kurobegwa Granite, was analyzed by zircon U–Pb together with geochemical analyses. It was revealed that the Kurobegawa Granite is a resurgent pluton that intruded at a shallow crustal level of ~ 6 km depth, following the caldera-forming ~ 1.55 Ma Jiigatake eruption. The rapid uplift and erosion of the pluton was caused not by the E–W compression but by resurgence. The pluton was formed mainly by resurgent magma culminated at ~ 1.0 Ma with preceding (~ 2.1–2.0 Ma) and protracted (~ 0.8–0.7 Ma) phases.

## Methods

### LA–ICP–MS U–Pb dating at CRIEPI

Approximately 1 kg of rock (mostly fresh) from each sample was crushed using a jaw crusher or a rock hammer to < 1 cm pieces followed by powdering in a disc mill. The powders were sieved using a disposable 250 µm cloth mesh and heavy minerals < 250 µm were concentrated by panning under running water. The concentrates were dried and further separated by standard magnetic and heavy liquid techniques. Some zircons were treated with acid (HF and HCl) to remove impurities. Zircons were handpicked and embedded in a PFA Teflon sheet and mostly unpolished surface was targeted for analyses.

Zircon U–Pb dating was performed at the Central Research Institute of Electric Power Industry (CRIEPI), using LA–ICP–MS (on a Thermo Fisher Scientific ELEMENT XR magnetic sector-field ICP–MS coupled to a New Wave Research UP-213 Nd–YAG laser) with experimental conditions following Ito^48^ (Supplementary Table S1). Samples were ablated in helium gas by pulses at a 10 Hz repetition rate. The focus of the laser beam was fixed at the sample surface throughout the data acquisition. Three different sets of laser ablation parameters were adopted: (1) 40 μm laser spot with 3–4 J/cm^2^ energy density, (2) 25 μm laser spot with 9–10 J/cm^2^ energy density, and (3) 30 μm laser spot with 7–8 J/cm^2^ energy density. The reason for changing experimental parameters (from 40 μm spot size to 25 μm and to 30 μm) was simply due to changes in laboratory protocols. Data were acquired in electrostatic scanning (E-scan) mode over 1080 mass scans during a 30 s background measurement, followed by a 30 s sample ablation and then a 45 s background (washout) measurement. ^235^U was calculated from ^238^U assuming ^238^U/^235^U = 137.818 (Hiess et al.^49^). Both unknowns and standards were measured in the same conditions.

Data for the first 10 s of ablation were omitted to avoid surface Pb contamination and signal instability, and the following 10 s of data were used to calculate U–Pb ages. The laser ablated to a depth of ~ 24 µm for 30 μm beam was during the 30 s analysis and therefore the U–Pb isotopic data is from approximately 8–16 µm depths within the zircon crystal.

Individual U–Pb ages were corrected for common-Pb using a modified ^207^Pb-based method^50^ using values from Stacey and Kramers^51^ and the measured ^207^Pb/^206^Pb. The modified ^207^Pb method employs both Th/U and Pa/U partitioning (*f*_Th/U_ and *f*_Pa/U_, respectively) for correcting initial ^230^Th and ^231^Pa disequilibrium in the zircon-magma system. Measured *f*_Th/U_ with 50% uncertainty and an assumed *f*_Pa/U_ of 3.36 ± 0.40 (Sakata^50^) were used for all age calculations. Data with a high (> 75%) common Pb contamination (*f*_206_%) or a high (> 70%) uncertainty were excluded for further analyses^16^ and the mean square weighted deviation (MSWD) is used as a statistical test of validity of weighted mean ages. Polished 91500 zircon^52^ was used as a primary geochronology standard with polished Plešovice^53^ zircon and unpolished Bishop Tuff^54^ zircon used as secondary reference materials.

U and Th concentrations were quantified by comparing counts of ^238^U and ^232^Th for the sample relative to the standard 91500, which is assumed to have homogeneous U and Th concentrations of 80 and 30 ppm respectively^52^, followed by a correction relative to NIST SRM 610 glass standard. No down-hole isotopic (Pb/U, Th/U) fractionation correction was performed because data from the same depth range (or time span) was used for standards and unknowns in each analysis. The result is shown in Supplementary Table S2.

The U–Pb ages from the Plešovice and the Bishop Tuff zircons were 339.3 ± 0.9 Ma and 0.75 ± 0.02 Ma (Supplementary Table S2), respectively, which are close to and in accordance with their reference ages of 337.13 ± 0.37 Ma^53^ and 0.767 ± 0.001 Ma^54^, respectively.

### Whole-rock composition analysis at CRIEPI

Whole-rock geochemical analyses were performed using the coarse (> 250 µm) fraction obtained during zircon separation. Approximately 20 g of coarse material was washed with distilled water and dried completely at 110 °C overnight. Rock powders were prepared using a tungsten-carbide mill and an agate-ball mill. Then, they were heated at 110 °C (24 h). Loss on ignition (LOI) was measured by weighing the sample before and after 750 °C (1 h) heating. Fused glass discs were prepared with a lithium tetraborate (Li_2_B_4_O_7_) flux in a 1:10 dilution ratio at 1050 °C.

Major element (Si, Ti, Al, Fe, Mn, Mg, Ca, Na, K, and P) compositions were determined by energy-dispersive spectrometry (EDS) and an X-ray fluorescence spectrometry (XRF) at CRIEPI. EDS spectra were produced using a Hitachi TM4000Plus scanning electron microscope (SEM) equipped with an Oxford AZtecOne EDS system, under an acceleration voltage of 15 kV and working distance of 10 mm. For the XRF analysis using SHIMADZU XRF-1500, the accelerating voltage was 20 kV, and the current was 90 mA. To monitor analytical precision, we analyzed ATHO-G and GOR128-G^55^ for EDS and JR-1 (Imai et al.^56^) for XRF as working standards during most analyses. The result is shown in Supplementary Table S4.

### Trace element analysis at CRIEPI

Multiple element analysis of whole rock and zircon were performed using LA–ICP–MS with experimental conditions shown in Ito^57^. Laser sampling was accomplished employing a 213 nm Nd–YAG laser connected to a Thermo Fisher Scientific ELEMENT XR sector field ICP–MS. Laser ablation was operated at a frequency of 10 Hz and an energy density of ~ 7 J/cm^2^, with a pit diameter of 100 μm for whole rock using the fused glass discs used for EDS analyses and 30 μm for the zircons used for U–Pb dating. Helium was applied as a carrier gas. Argon was used as the make-up gas and mixed with the carrier gas via a T-connector before entering the ICP. Samples and reference materials were analyzed for the 35 mass numbers listed in Ito^57^. Each analysis incorporated a background acquisition of 30 s (gas blank) followed by a 30 s data acquisition and a 45 s washout. Data were obtained at a 10–20 s (40–50 s from the start) time span. Corrections were made for mass bias drift which was evaluated by reference to the standard glass NIST 613. Element concentrations were obtained by normalizing count rates for each analyzed element to those for SRM 613 glass standard^55^ and Si, assuming SiO_2_ to be stoichiometric with a concentration of 32.7 wt% in zircon and 71.9 wt% in SRM613 glass^55^. P, K, Ca, Ti, Fe and Th were monitored in this study to evaluate possible involvements of mineral inclusions (apatite, K-feldspar, plagioclase, titanite, pyrite and monazite, respectively) in zircons or to monitor spikes. As a check of reproducibility, precision and accuracy, NIST 610 glass was also analyzed. Moreover, 91500 zircon^55^, Bishop Tuff zircon^58^, MAD-559 zircon^59^ were analyzed as secondary zircon standards and ATHO-G^55^ glass was analyzed as secondary glass standard in every 5–10 unknown measurements. The results are shown in Supplementary Table S4 and Supplementary Fig. S5a for whole rock and Supplementary Table S5 and Supplementary Fig. S6 for zircon.

### Trace element analysis at Akita University

Whole rock trace element compositions of powdered rock samples were determined by 7700× Quadrupole ICP–MS (Agilent, USA) at Akita University. The method is the same as Fukuyama et al.^60^. The granites contain acid resistant minerals such as zircon, thus samples were dissolved by alkali fusion after acid digestion. Geological reference material JB-1a and JG-1a were also analyzed at the same time to verify the accuracy of analysis. Samples were sequentially dissolved with concentrated HF plus trace amounts of concentrated HNO_3_, and 7 M HNO_3_ on a hot plate. Then the sample solutions were centrifuged. The solutions without residue were transferred into measuring flasks. Remaining solutions with residue were transferred in Pt crucibles for fusion with 0.1 g anhydrous Na_2_CO_3_ flux. After fusion on the gas burner, fused melts are dissolved with 7 M HNO_3_ and finally combined with residue free sample solutions in the measuring flask. Matrix-matched calibration standard with 10 ng/g and 100 ng/g of multi-element standard solution ICP-MS-68A and ICP-MS-68B (High Purity Standards, USA), respectively were prepared. Rhodium single element standard (High Purity Standards, USA) is added on-line to both standards and samples as an internal standard. The result is shown in Supplementary Table S4 and Supplementary Fig. S5b.

### Zircon hafnium isotope analysis at Akita University

After the U–Pb dating of zircons, Hf isotopes were determined on the same spots using a NWR 193 UC excimer laser ablation system attached to a Nu Plasma II multi-collector ICP-MS at Akita University. Instrument condition is shown in Supplementary Table S13. The analytical method is the same as Ogasawara et al.^61^. The result is shown in Supplementary Table S14 and Supplementary Fig. S7.

### Estimation of the volume of the Jiigatake Volcanics

Although the volume of the Jiigatake Volcanics was unknown^46^, it is estimated here assuming the following parameters. First, we assume that the two voluminous eruptions (Jiigatake and Hotaka eruptions) occurred from calderas that are delineated by Jiigatake–Kurobegawa and Hotaka–Takidani complexes (Fig. 1b). The corresponding caldera sizes are estimated to be ~ 160 km^2^ and ~ 80 km^2^, respectively. Second, we assume the volume of eruptions corresponds to the caldera size, because the two eruptions occurred in similar tectonic settings. Since the volume of Hotaka Andesite is estimated to be 750 km^3^ (Oikawa^46^), the volume of the Jiigatake Volcanics is calculated to be 1500 km^3^.

However, a 1500 km^3^ eruption requires an extraction of > 9 km thick magma for the caldera size of 160 km^2^, which seems unrealistic. We assume an extraction of ~ 2 km thick magma as a plausible estimate similar to the estimate of Long Valley Caldera, USA^62^. Therefore, we tentatively assume the volume of the Jiigatake Volcanics to  be ~ 300 km^3^. We assume that the volume of Hotaka Andesite (750 km^3^, summing 1.76 Ma 400 km^3^ and 1.75 Ma 350 km^3^ eruptions^46^) may be overestimated because it is exceptionally large compared to other calc-alkaline volcanic eruptions of similar size calderas in Japan (Supplementary Fig. S9; Supplementary Table S15). Alternatively, Gualda et al.^63^ showed that supereruption-forming magma bodies are ephemeral, being stored for no more than centuries to a few millennia before eruption, suggesting perhaps the 750 km^3^ volume is realistic.

## Supplementary Information

Supplementary Information 1.

Supplementary Information 2.

## Data Availability

Data are available in an Excel file as Supplementary data.

## References

[1] Hildreth W (2004). Volcanological perspectives on long Valley, Mammoth Mountain, and Mono Craters: Several contiguous but discrete systems. J. Volcanol. Geotherm. Res..

[2] Bachmann O, Miller CF, de Silva S (2007). The volcanic–plutonic connection as a stage for understanding crustal magmatism. J. Volcanol. Geotherm. Res..

[3] Coleman DS, Gray W, Glazner AF (2004). Rethinking the emplacement and evolution of zoned plutons: Geochronologic evidence for incremental assembly of the Tuolumne Intrusive Suite, California. Geology.

[4] Glazner AF, Bartley JM, Coleman DS, Gray W, Taylor RZ (2004). Are plutons assembled over millions of years by amalgamation from small magma chambers?. GSA Today.

[5] Davis JW, Coleman DS, Gracely JT, Gaschnig R, Stearns M (2012). Magma accumulation rates and thermal histories of plutons of the Sierra Nevada batholith, CA. Contrib. Mineral. Petrol..

[6] Matsubara M, Hirata N, Sakai S, Kawasak I (2000). A low velocity zone beneath the Hida Mountains derived from dense array observation and tomographic method. Earth Planets Space.

[7] Waite GP, Moran SC (2009). Vp structure of Mount St. Helens, Washington, USA, imaged with local earthquake tomography. J. Volcanol. Geotherm. Res..

[8] Chu R, Helmberger DV, Sun D, Jackson JM, Zhu L (2010). Mushy magma beneath Yellowstone. Geophys. Res. Lett..

[9] Jaxybulatov K (2014). A large magmatic sill complex beneath the Toba caldera. Science.

[10] Flinders AF (2018). Seismic evidence for significant melt beneath the Long Valley Caldera, California, USA. Geology.

[11] Rasht-Behesht M, Huber C, Mancinelli NJ (2020). Detectability of melt-rich lenses in magmatic reservoirs from teleseismic waveform modeling. J. Geophys. Res. Solid Earth.

[12] Nyst M, Nishimura T, Pollitz FF, Thatcher W (2006). The 1923 Kanto earthquake re-evaluated using a newly augmented geodetic data set. J. Geophys. Res. Solid Earth.

[13] Harayama S, Ohyabu K, Miyama Y, Adachi H, Shukuwa R (2003). Eastward tilting and uplifting after the late early Pleistocene in the eastern-half area of the Hida Mountain Range. Q. Res. (Daiyonki-Kenkyu).

[14] Smith RL, Bailey RA (1968). Resurgent cauldrons. Geol. Soc. Am. Mem..

[15] Harayama S (2015). Vertically turned quaternary collapsed caldera and Kurobegawa Granite complex, exposed around the Mt. Kashimayari and Mt. Jii, Northern Japan Alps: A typical example of tilting mountain uplift under contraction tectonics. J. Geol. Soc. Jpn..

[16] Ito H (2013). Earth’s youngest exposed granite and its tectonic implications: the 10–0.8 Ma Kurobegawa Granite. Sci. Rep..

[17] Ito H, Spencer CJ, Danišík M, Hoiland CW (2017). Magmatic tempo of Earth’s youngest exposed plutons as revealed by detrital zircon U-Pb geochronology. Sci. Rep..

[18] Spencer CJ (2019). Rapid exhumation of Earth’s youngest exposed granites driven by subduction of an oceanic arc. Geophys. Res. Lett..

[19] Harayama S, Wada H, Yamaguchi Y (2003). Quaternary and pliocene granites in the Northern Japan Alps. Hutton symposium V, field guidebook, trip A1. SGeol. Surv. Jpn. Interim-Rep..

[20] Bando M, Bignall G, Sekine K, Tsuchiya N (2003). Petrography and uplift history of the quaternary Takidani Granodiorite: Could it have hosted a supercritical (HDR) geothermal reservoir?. J. Volcanol. Geotherm. Res..

[21] Hartung E (2017). Evidence for residual melt extraction in the Takidani Pluton, Central Japan. J. Petrol..

[22] Ito H, Tamura A, Morishita T, Arai S (2012). Timing of some plutonic intrusions and tectonics in the Hida Mountain Range: An application of LA-ICP-MS U-Pb dating on zircons. J. Geol. Soc. Jpn..

[23] Wada H, Harayama S, Yamaguchi Y (2004). Mafic enclaves densely concentrated in the upper part of a vertically zoned felsic magma chamber: The Kurobegawa granitic pluton, Hida Mountain Range, central Japan. Bull. Geol. Soc. Amer..

[24] Ishizawa K (1982). Geology of the igneous rocks in the Mt. Kashimayarigatake–Mt. Eboshidake area, Hida Mountains, central Japan. J. Geol. Soc. Jpn..

[25] Kawakami T (2021). Solidification depth and crystallization age of the Shiaidani Granodiorite: Constraints to the average denudation rate of the Hida Range, central Japan. Island Arc.

[26] Watson EB, Harrison TM (1983). Zircon saturation revisited: Temperature and composition effects in a variety of crustal magma types. Earth Planet. Sci. Lett..

[27] Molina JF (2021). A reassessment of the amphibole-plagioclase NaSi–CaAl exchange thermometer with applications to igneous and high-grade metamorphic rocks. Am. Mineral..

[28] Holland T, Blundy J (1994). Non-ideal interactions in calcic amphiboles and their bearing on amphibole-plagioclase thermometry. Contrib. Mineral. Petrol..

[29] Mutch EJF, Blundy JD, Tattitch BC, Cooper FJ, Brooker RA (2016). An experimental study of amphibole stability in low-pressure granitic magmas and a revised Al-in-hornblende geobarometer. Contrib. Mineral. Petrol..

[30] Erdmann S (2019). Titanite: A potential solidus barometer for granitic magma systems. C. R. Geosci..

[31] Schmidt MW, Thompson AB (1996). Epidote in calc-alkaline magmas: An experimental study of stability, phase relationships, and the role of epidote in magmatic evolution. Am. Mineral..

[32] Bea F, Morales I, Molina JF, Montero P, Cambeses A (2021). Zircon stability grids in crustal partial melts: Implications for zircon inheritance. Contrib. Mineral. Petrol..

[33] Huber C, Townsend M, Degruyter W, Bachmann O (2019). Optimal depth of subvolcanic magma chamber growth controlled by volatiles and crust rheology. Nat. Geosci..

[34] Harayama S, Takahashi M, Shukuwa R, Itaya T, Yagi K (2010). High-temperature hot springs and quaternary Kurobegawa Granite along the Kurobegawa River. J. Geol. Soc. Jpn..

[35] Ludwig KR (2012). User’s Manual for Isoplot 3.75: A geochronological toolkit for Microsoft Excel. Berkeley Geochronol. Center Spec. Pub..

[36] Vermeesch P (2018). IsoplotR: A free and open toolbox for geochronology. Geosci. Front..

[37] Kennedy B, Wilcock J, Stix J (2012). Caldera resurgence during magma replenishment and rejuvenation at Valles and Lake City calderas. Bull. Volcanol..

[38] de Silva SL, Mucek A, Gregg P, Pratomo I (2015). Resurgent Toba–field, chronologic, and model constraints on time scales and mechanisms of resurgence at large calderas. Front. Earth Sci..

[39] Corradino M (2021). Resurgent uplift at large calderas and relationship to caldera-forming faults and the magma reservoir: New insights from the Neapolitan Yellow Tuff caldera (Italy). J. Volcanol. Geotherm. Res..

[40] Di Vito MA (2016). Magma transfer at Campi Flegrei caldera (Italy) before the last 1538 AD eruption. Sci. Rep..

[41] Koulakov I (2016). The feeder system of the Toba supervolcano from the slab to the shallow reservoir. Nat. Commun..

[42] Lipman PW, Bachmann O (2015). Ignimbrites to batholiths: Integrating perspectives from geological, geophysical, and geochronological data. Geosphere.

[43] Karakas O, Degruyter W, Bachmann O, Dufek J (2017). Lifetime and size of shallow magma bodies controlled by crustal-scale magmatism. Nat. Geosci..

[44] Otofuji Y (1996). Large tectonic movement of the Japan arc in the late Cenozoic times inferred from paleomagnetism: Review and synthesis. Island Arc.

[45] Sawaki Y, Asanuma H, Abe M, Hirata T (2020). U-Pb ages of granitoids around the Kofu basin: Implications for the Neogene geotectonic evolution of the South Fossa Magna region, central Japan. Island Arc.

[46] Oikawa T (2003). The spatial and temporal relationship between uplifting and magmatism in the Hida Mountain Range. Central Japan. Quat. Res. (Daiyonki-Kenkyu).

[47] Tatsumi Y (2020). Contrasting volcano spacing along SW Japan arc caused by difference in age of subducting lithosphere. Sci. Rep..

[48] Ito H (2020). Magmatic history of the Oldest Toba Tuff inferred from zircon U-Pb geochronology. Sci. Rep..

[49] Hiess J, Condon DJ, McLean N, Noble SR (2012). ^238^U/^235^U systematics in terrestrial U-bearing minerals. Science.

[50] Sakata S (2018). A practical method for calculating the U-Pb age of Quaternary zircon: Correction for common Pb and initial disequilibria. Geochem. J..

[51] Stacey JS, Kramers JD (1975). Approximation of terrestrial lead isotope evolution by a two-stage model. Earth Planet. Sci. Lett..

[52] Wiedenbeck M (2004). Further characterisation of the 91500 zircon crystal. Geostand. Geoanal. Res..

[53] Sláma J (2008). Plešovice zircon—A new natural reference material for U-Pb and Hf isotopic microanalysis. Chem. Geol..

[54] Crowley JL, Schoene B, Bowring SA (2007). U-Pb dating of zircon in the Bishop Tuff at the millennial scale. Geology.

[55] Jochum KP, Stoll B, Sylvester P (2008). Reference materials for elemental and isotopic analysis by LA-(MC)-ICP-MS: successes and outstanding needs. Laser Ablation-ICP-MS in the Earth Sciences, Current Practices and Outstanding Issues. Mineralogical Association of Canada (MAC) Short Course Series.

[56] Imai N, Terashima S, Itoh S, Ando A (1995). 1994 compilation of analytical data for minor and trace elements in seventeen GSJ geochemical reference samples 'Igneous Rock Series'. Geostand. Newslett..

[57] Ito H (2018). Bishop Tuff as a reference material for U-Pb dating and elemental analyses. Fission-Track Newslett..

[58] Reid MR, Vazquez JA, Schmitt AK (2011). Zircon-scale insights into the history of a Supervolcano, Bishop Tuff, Long Valley, California, with implications for the Ti-in-zircon geothermometer. Contrib. Mineral. Petrol..

[59] Coble MA (2018). Trace element characterisation of MAD-559 zircon reference material for ion microprobe analysis. Geostand. Geoanal. Res..

[60] Fukuyama M, Kawamoto T, Ogasawara M (2017). Chemical composition of fluid inclusions in the Yorii jadeite-quartz rocks from the Kanto Mountains, Japan. J. Min. Pet. Sci..

[61] Ogasawara M, Fukuyama M, Siddiqui RH, Zhao Y (2019). Origin of the Ordovician Mansehra granite in the NW Himalaya, Pakistan: Constraints from Sr-Nd isotopic data, zircon U-Pb age and Hf isotopes. Geol. Soc. Lond. Spec. Pub..

[62] Hildreth W, Fierstein J, Calvert A (2017). Early postcaldera rhyolite and structural resurgence at Long Valley Caldera, California. J. Volcanol. Geotherm. Res..

[63] Gualda (2018). Climbing the crustal ladder: Magma storage-depth evolution during a volcanic flare-up. Sci. Adv..

