# Supplementary material for: The Quaternary Kurobegawa Granite: an example of a deeply dissected resurgent pluton
Source: Sci Rep. 2021 Nov 11;11:22059. doi: 10.1038/s41598-021-01562-2 (PMC8585956; doi:10.1038/s41598-021-01562-2)
Supplement: Supplementary file 1 — Supplementary Information 1. [file 41598_2021_1562_MOESM1_ESM.pdf]

Supplementary Information

The Quaternary Kurobegawa Granite: an example of a deeply dissected resurgent pluton

Hisatoshi Ito[1], Yoshiko Adachi[1], Aitor Cambeses[2], Fernando Bea[2], Mayuko Fukuyama[3], Koji Fukuma[4], Ryuji Yamada[5], Takashi Kubo[6], Mami Takehara[7], Kenji Horie[7, 8]

[1]*Central Research Institute of Electric Power Industry, Chiba 270-1194, Japan*
[2]*Department of Mineralogy and Petrology, University of Granada, Granada 18002, Spain*
[3]*Graduate School of Engineering Science, Akita University, Akita 010-8502, Japan*
[4]*Department of Environmental System Science, Doshisha University, Kyotanabe 610-0394, Japan*
[5]*National Research Institute for Earth Science and Disaster Resilience, Ibaraki 305-0006, Japan*
[6]*Asahi Town Board of Education, Toyama 939-0743, Japan*
[7]*National Institute of Polar Research, Tokyo 190-8518, Japan*
[8]*Department of Polar Sciences, The Graduate University for Advanced Studies, SOKENDAI, Tokyo 190-8518, Japan*

Contents:

SHRIMP U-Pb dating at Granada University
Geochemical analyses at Granada University
References

Figures S1–S9

SHRIMP U-Pb dating at Granada University

A granite fraction from sample KRW (KRW-3) (Fig. 3a, d) was processed for zircon SHRIMP U-Pb dating. Zircon was separated using panning, first in water and then in ethanol. This was followed by magnetic extraction of Fe-rich minerals with a Nd magnet. Finally, zircons were handpicked using a binocular microscope. The zircons were cast in epoxy "megamounts" (i.e., 35 mm epoxy discs) which are fixed on the front of a mount holder so that there are no metallic parts or surface discontinuities near the samples that might fractionate extraction of secondary ions. The minerals were carefully studied with optical (reflected and transmitted light) and scanning electronic microscopy (backscattering and cathodoluminescence) prior to SHRIMP analyses with the IBERSIMS SHRIMP IIe/mc ion microprobe.

Zircons were analyzed for U-Pb following the method described by Williams and Claesson[64]. The mount was coated with a ~12 nm thick gold layer. Each spot was rastered with the primary beam for 120 s prior to analysis and then analyzed with 6 scans following the isotope peak sequence $^{196}Zr_2O$, ^{204}Pb, $^{204.1}$background, ^{206}Pb, ^{207}Pb, ^{208}Pb, ^{238}U, ^{248}ThO, ^{254}UO. Each peak was measured with the following counting times: 2 s for mass 196; 5 s for masses 238, 248, and 254; 15 s for masses 204, 206, and 208; and 20 s for mass 207. The primary beam, composed of $^{16}O_2^-$, was set to an intensity of about 5 nA, using a 120 microns Kohler aperture, which generated 17 × 20 μm elliptical spots on the target. The secondary beam exit slit was fixed at 80 μm, achieving a mass resolution of about 5000 at 1% peak height. All calibration procedures were performed on the standards included on the same mount. Mass calibration was done on the REG zircon (ca. 2.5 Ga, very high U, Th and common lead content). The analytical session started by measuring the SL13 zircon[65], which was used as a concentration standard (238 ppm U). TEMORA-2 zircon (416.8 ± 1.1 Ma)[66], was used as an isotope ratios standard and measured repeatedly after every 4 unknown analyses. Data

reduction was done with the SHRIMPTOOLS software (available from www.ugr.es/~fbea), which is a new implementation of the PRAWN software originally developed for the SHRIMP. Errors are reported at the 95% confidence interval (C.I.: ~2 σ). Standard errors (95% C.I.) on the 37 replicates of the TEMORA standard measured during the analytical session were ± 0.35% for $^{206}Pb/^{238}U$ and ± 0.83% for $^{207}Pb/^{206}Pb$. Due to the extremely young ages of KWR sample, we applied the ^{230}Th correction[67,68] considering a Th/U ratio of 29.0 from whole-rock composition of sample KWR-3 (Supplementary Table S4). The results are shown in Supplementary Table S3.

Sample KRW-3 contained abundant zircon grains with euhedral to subhedral elongated prims with bipyramidal termination morphologies (300 μm × 150 μm) (Supplementary Fig. S3). Most grains are colourless and transparent, although some are pinkish translucent crystals.

Twenty-one U-Pb measurements on 14 different zircons yielded moderate concentrations of U (254–932 ppm) and Th (127–812 ppm) with Th/U 0.34–0.95 (Supplementary Table S3). All analyzed data are plotted along a discordant line with a lower interception providing an age of 0.79 ± 0.12 Ma (MSWD = 19.26). The weighted means (errors reported at 2σ) of the 207-corrected $^{238}U/^{206}Pb$ age yielded an age of 0.77 ± 0.04 Ma (MSWD = 2.25) (Supplementary Fig. S3).

Geochemical analyses at Granada University

Mineral chemistry

Major element analyses of minerals were obtained by SEM and EDS with a Zeiss DSM-950 scanning microscope equipped with a Link Isis series 300 Analytical Pentafet system, operated at 20 kV and 1–2 nA bean current. Natural and synthetic standards were employed in the analyses of mineral major elements.

Precision was better than ± 1.5 proportion for analyte concentrations of 1 wt% proportion. Minerals were normalized to total 100 wt% and 8 oxygen atoms for plagioclase and alkali feldspar, total 95 wt% and 22 oxygen atoms for biotite, total 97 wt% and 23 oxygen atoms for amphibole and total 100 wt% and 5 oxygens atoms for sphene (The results are shown in Supplementary Tables S6–S10).

Three populations of amphiboles are distinguished according to textural-chemical features in MME: Amp-1 has magnesiohastingsite compositions and it forms core and mantle of large crystals characterized by low content in total Fe (1.606–2.284 atoms per formula unit: apfu) and high total Al (1.878–2.352 apfu) and Ti (0.196–0.309 apfu). Amp-2, hastingsite, overgrowths Amp-1 and it trends away from the composition of Amp-1, being richer in total Fe (2.362–2.784 apfu) and similar content in Ti (0.211–0.308 apfu) and total Al (1.835–2.276 apfu). Amp-3, edenite to magnesiohornblende, are small crystals and the aforementioned thin external patchy rims on all the other amphibole type. This amphibole is characterized by high total Fe (2.348–2.889 apfu) and low total Al (0.863–1.768 apfu) and Ti (0.017–0.251 apfu). Biotite (Mg#, $Mg/(Mg+Fe^{2+})$ = 0.39–0.43) is less abundant than amphibole and it shows remarkable variation in total Al (2.321–2.548 apfu). Plagioclase has complex textural and compositional features. Some grains contain spongy and reabsorbed crystals, Pl-1, being bytownite $An_{70–90}$ as inclusion in larger crystal of andesine to oligoclase, Pl-2, with $An_{17–44}$. Pl-3, appears surrounding Pl-2 and forming subhedral tabular crystals. Pl-3 is quite varied from labradorite cores to oligoclase rims with $An_{16–70}$. K-feldspar is present as inclusions in Pl-2 and as interstitial grains yielding a similar compositional range with $Or_{83–100}$. Sphene is commonly included in Amp-2 and Amp-3 and is characterized by $Al+Fe^{3+}$ = 0.194–0.286 apfu and Ti = 0.783–0.870 apfu.

Amphibole in host granite is very scarce and it is restricted to small grains included in plagioclase, with compositional features of Amp-2, and small interstitial grains, the latter matching with Amp-3. Biotite is the

main hydrated ferromagnesian mineral in granite with a homogeneous composition (Mg# = 42–45) and is richer in total Al (2.526–2.604 apfu) and poorer in Ti (0.393–0.510 apfu) than biotite from MMEs. There are two kinds of plagioclases in host granites: Pl-4 represents large megacryts with slightly zonation from core to rim from andesine to oligoclase with $An_{11–37}$. Pl-5 are small grains with a large compositional zonation from labradorite to oligoclase with $An_{16–62}$. K-feldspar is present as anhedral inclusions in Pl-4 and as interstitial grains with same composition of $Or_{80–100}$.

Whole-rock composition

Whole rock major element determinations were performed by XRF, after fusion with lithium tetraborate (Supplementary Table S4). Typical precision was better than ±1.5% for an analyte concentration of 10 wt%. Zirconium was determined by X-ray fluorescence on the same disks, with a precision better than ± 4% for 100 ppm Zr. Trace element determinations were done by ICP-mass spectrometry (ICP-MS) after HNO_3+HF digestion of 0.1000 g of sample powder in a Teflon-lined vessel at ~180 °C and 200 psi for 30 min, evaporation to dryness, and subsequent dissolution in 100 ml of 4 vol% HNO_3. Instrument measurements were carried out in triplicate with a PE SCIEX ELAN- 8000 spectrometer using rhodium as an internal standard. Precision, as determined from standards WS-E, BR and AGV run as unknowns, was better than ± 2% and ± 5% for analyte concentrations of 50 and 5 ppm, respectively. Samples for Sr and Nd isotope analysis (0.1000 g) were digested with HNO_3+HF in a Teflon-lined vessel at 200 psi. The elements were separated with ion-exchange resins, and the Sr and Nd isotope ratios were determined by thermal ionization mass spectrometry with a Finnigan Mat 262. All reagents were ultra clean. Normalization values were $^{86}Sr/^{88}Sr$ = 0.1194 and $^{146}Nd/^{144}Nd$ = 0.7219. Blanks were 0.6 and 0.09 ng for Sr and Nd, respectively. The external precision (2σ), estimated by analyzing 10 replicates of the standard WS-E[69], was better than ±

0.003% for $^{87}Sr/^{86}Sr$ and ± 0.0015% for $^{143}Nd/^{144}Nd$. $^{87}Rb/^{86}Sr$ and $^{147}Sm/^{144}Nd$ were directly determined by ICP-MS following the method developed by Montero and Bea[70], with a precision better than ± 1.2% and ± 0.9% (2σ), respectively. Simple binary magma mixing model for major, trace elements and isotopes were calculated according to Faure and Mesning[71]. We consider sample KRW-1 as mafic end-member and sample KRW-3 as felsic end-member. Mixing products were calculated for every 10% of weight fractions for each end-member (Supplementary Table S4).

MMEs are subalkaline (SiO_2 = 58–61 wt%, Na_2O+K_2O = 5.3–5.9 wt%), tholeiitic (FeO_T/MgO = 2.6–3.2) and metaluminous (ASI, molar (Al_2O_3)/(CaO + Na_2O + K_2O) = 0.90–0.95). Major element concentrations in MMEs are in the range Al_2O_3 = 17.6–18.2 wt%, TiO_2 = 0.6–0.8 wt%, FeO_T = 7–8 wt%, MgO = 2.1–2.7 wt%, CaO = 5.6–6.4 wt%, Na_2O = 3.4–3.7 wt%, K_2O = 1.8–2.2 wt% and P_2O_5 = 0.3–0.4 wt% (Supplementary Table S4). The MMEs show chondrite-normalised enrichment in light rare earth element (LREE) relative to heavy rare earth element (HREE), LaN/LuN = 5.6–6.3, and negative Eu anomalies, Eu/Eu^* = 0.73–0.88 (Supplementary Fig. S4c). The isotopes from MME have the following isotopic composition: $\varepsilon Nd_{0.8\ Ma}$ = 0.2 and $^{87}Sr/^{86}Sr_{0.8\ Ma}$ = 0.705375 (Supplementary Table S4).

The host granites have a subalkaline composition (SiO_2 = 71–72 wt%, Na_2O+K_2O = 7.2 wt%), slightly peraluminous (ASI = 1.02) and calc-alkaline (FeO_T/MgO = 3.5–3.6). Major element concentrations in host granites are Al_2O_3 = 14.8–15.1 wt%, TiO_2 = 0.3 wt%, FeO_T = 3 wt%, MgO = 0.7 wt%, CaO = 2.7 wt%, Na_2O = 3.4 wt%, K_2O = 3.7 wt% and P_2O_5 = 0.12 wt% (Supplementary Table S4). They are enriched in LREE relative to HREE, LaN/LuN = 14.2–15.6, with positive Eu anomalies, Eu/Eu^* = 1.1–1.2. In comparison to MME, the host granites have $\varepsilon Nd_{0.8\ Ma}$ = -1.0 and $^{87}Sr/^{86}Sr_{0.8\ Ma}$ = 0.705648.

In the intermingled zone it is possible to recognize complex textural relationship of main phases from MME´s and host granite, well indicated amphibole, biotite and plagioclase. It suggests an interaction and mixing of contrasted magmas and subsequent reequilibration process of main phases and to crystallise new phases derived from hybrid magma.

Thermobarometric estimations

The crystallization temperatures were determined using: 1) the zircon-saturation thermometer of Borisov and Aranovich[72] for MMEs and Bohenke et al.[73] for intermediate to felsic compositions, and 2) the apatite-saturation thermometer[74], with a correction proposed by Bea et al.[75] for the peraluminous compositions for whole-rock data (Fig. 4a).

We also carried out amphibole (Amp)-plagioclase (Pl) thermobarometric estimations in MMEs samples. Due to complex textural and compositional variations observed in amphibole and plagioclase, we carefully selected Amp-Pl pairs to ensure the equilibrium between these two phases. We consider four kinds of pairs: group 1) Amp-1 (Al: 2.133–2.254 apfu)- Pl-1 (anorthite, An: 0.70–0.76); group 2) Amp-2 (Al: 1.963–2.276 apfu)- Pl-3 (An: 0.64–0.69); group 3) Amp-3 (Al: 1.294–1.592 apfu)- Pl-3 (An: 0.28–0.37); and group 4) Amp-4 (Al: 0.862–1.015 apfu)- Pl-3 (An: 0.16–0.23) (Fig. 4b). We used the Al-in-hornblende barometer[29] as MMEs samples bear the buffering assemblage of quartz + plagioclase + K feldspar + amphibole + titanite. We also used Al in sphene barometer[30] calibrated for granitic rocks in equilibrium with amphibole, plagioclase, K-feldspar, quartz, biotite, and magnetite ± ilmenite. For temperature estimations, we considered the new three thermometers of Molina et al.[27] for groups 1 and 2 and the edenite-albite-richerterite-anorthite (Ed-Ab-Rich-An) Amp-Pl thermometer of Holland and Blundy[28] for groups 3 and 4. The thermometer of Molina et al.[27] was not used in the latter groups because the selected

Amp-Pl compositional pairs lie outside the field of experimental Amp-Pl pairs used for calibration of Molina et al.[27]. All calculations are available in Supplementary Tables S10–S12.

The zircon saturation temperatures for the KRW sample and Wada et al.[23] data are, in general, lower than those of apatite saturation (Fig. 4a). MMEs have low zircon saturation temperatures of 648–688 ºC, and for apatite, 904–950 ºC. The host granites have higher zircon saturation temperatures, 692–714 ºC; although similar apatite saturation temperatures, 919–928 ºC. The intermingled samples have similar zircon (694–745 ºC) and apatite (910–950 ºC) saturation temperatures with host granites.

For MMEs the Amp-Pl temperature estimates are 894–924 ºC for group 1, 734–851 ºC for group 2, 799–852 ºC for group 3 and 723–766 ºC for group 4 (Fig. 4b). Amphibole-only pressure estimates cluster around 5.7–6.3 kbar and 5.0–6.4 kbar for groups 1 and 2, respectively (Fig. 4b). Estimated pressure of Al in sphene yields a range of 4.8–6.4 kbar, similar with amphibole barometer for groups 1 and 2 (Fig. 4b). However, groups 3 and 4 have progressive lower pressure range of 2.6–3.6 to 1.5–1.9 kbar, respectively (Fig. 4b). The high pressure and temperature conditions of groups 1 and 2 can be interpreted as early crystallized phases when MMEs magma was rising up towards shallow levels. By contrast, the lower pressure range of groups 3 and 4 indicates the depth conditions, 5–7 km, where MMEs magma interacted with host granite magma chamber.

References

64. Williams, I. S. & Claesson, S. Isotopic evidence for the Precambrian provenance and Caledonian metamorphism of high grade paragneisses from the Seve Nappes, Scandinavian Caledonides. *Contrib. Mineral. Petrol.* **97**, 205–217 (1987).

65. Claoue-Long, J., Compston, W., Roberts, J. & Fanning, C. M. Two carboniferous ages: a comparison of SHRIMP zircon dating with conventional zircon ages & $^{40}Ar/^{39}Ar$ analysis. In: Berggren, W. A., Kent, D. V., Aubry, M. P., Hardenbol, J. (eds) Geochronology, Time Scales & Stratigraphic Correlation. *SEPM Special Publication* **54**, 1–22 (1995).

66. Black, L. P. et al. TEMORA 1: a new zircon standard for Phanerozoic U–Pb geochronology. *Chemical geology* **200**, 155–170 (2003).

67. Williams, R. W. & Gaffney, A. M. ^{230}Th-^{234}U model ages of some uranium standard reference materials. *Proc. Radiochim. Acta* **1**, 31–35 (2011).

68. Rojas-Agramonte, Y. et al. Ancient xenocrystic zircon in young volcanic rocks of the southern Lesser Antilles island arc. *Lithos* **290**, 228–252 (2017).

69. Govindaraju, K., Potts, P. J., Webb, P. C. & Watson, J. S. 1994 Report on Whin Sill Dolerite WS-E from England and Pitscurrie Micrograbbro PM-S from Scotland: assessment by one hundred and four international laboratories. *Geostandards Newslett.* **18**, 211–300 (1994).

70. Montero, P. & Bea, F. Accurate determination of $^{87}Rb/^{86}Sr$ and $^{147}Sm/^{144}Nd$ ratios by inductively-coupled-plasma mass spectrometry in isotope geoscience: an alternative to isotope dilution analysis. *Analytica Chimica Acta* **358**, 227–233 (1998).

71. Faure, G. & Mensinig, T. M. Isotopes Principles and Applications, third ed. Willey and Sons, Hoboken, New Jersey (2005).

72. Borisov, A. & Aranovich, L. Zircon solubility in silicate melts: New experiments and probability of zircon crystallization in deeply evolved basic melts. *Chemical Geology* **510**, 103–112 (2019).

73. Boehnke, P., Watson, E. B., Trail, D., Harrison, T. M. & Schmitt, A. K. Zircon saturation re-visited. *Chemical Geology* 3**51**, 324–334 (2013).

74. Harrison, T. M. & Watson, E. B. The behavior of apatite during crustal anatexis: Equilibrium and kinetic considerations. *Geochim. Cosmochim. Acta* **48**, 1467–1477 (1984).

75. Bea, F., Fershtater, G. & Corretgé, L. G. The geochemistry of phosphorus in granite rocks and the effect of aluminium. *Lithos* **29**, 43–56 (1992).

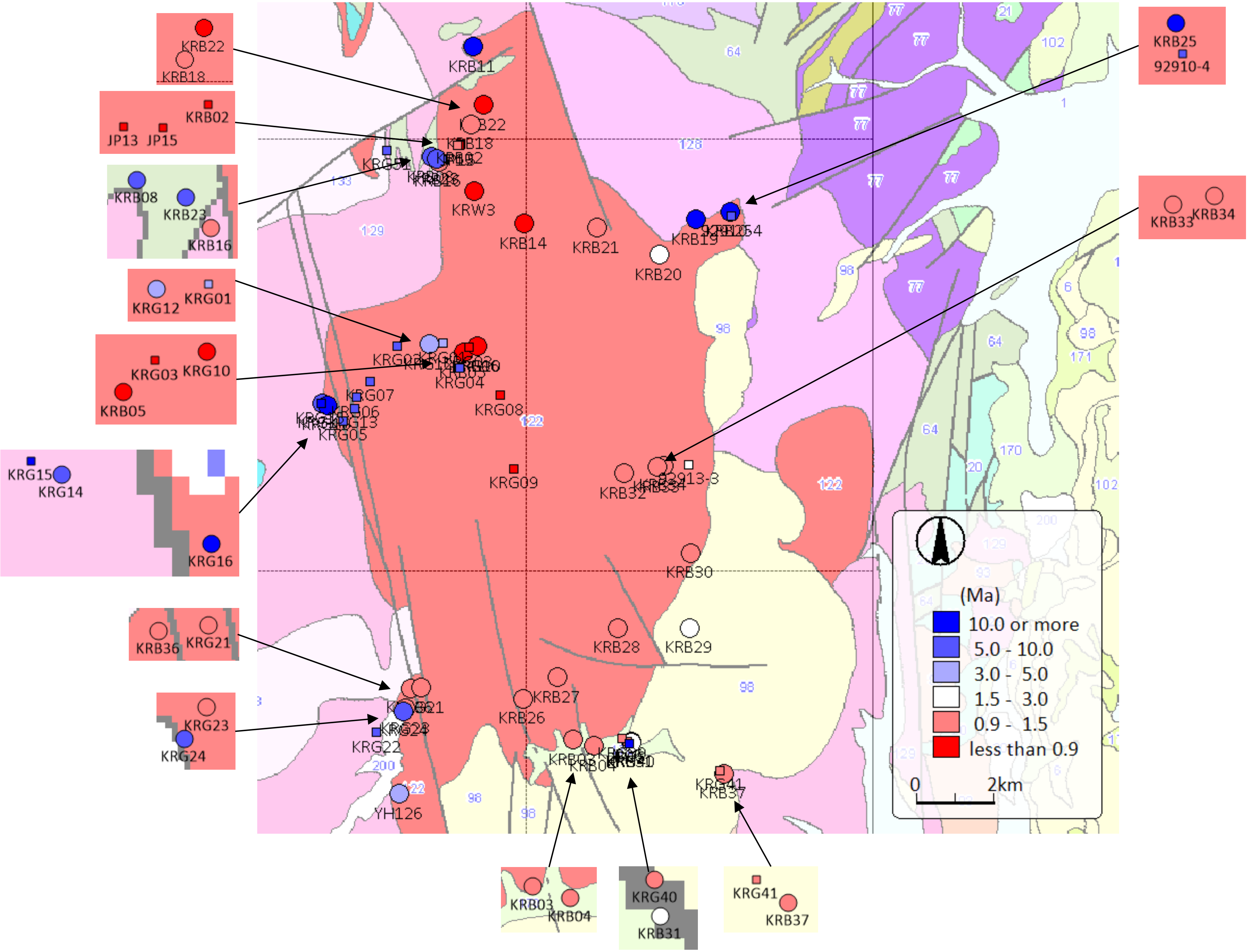

Supplementary Fig. S1. Geological map in and around the Kurobegawa Granite showing the sample locations, sample name and zircon U-Pb ages. Large circles and small squares are ages in this study and the literature[16-18], respectively. Geological map is modified from Seamless digital geological map of Japan 1: 200,000 (Geological Survey of Japan, AIST) using MANDARA10 (version 10.0.1.5) (https://ktgis.net/mandara/). Some overlapped sample names are shown as magnified maps for clarity. Geological legends are from https://gbank.gsj.jp/seamless/legend_shosai.html.

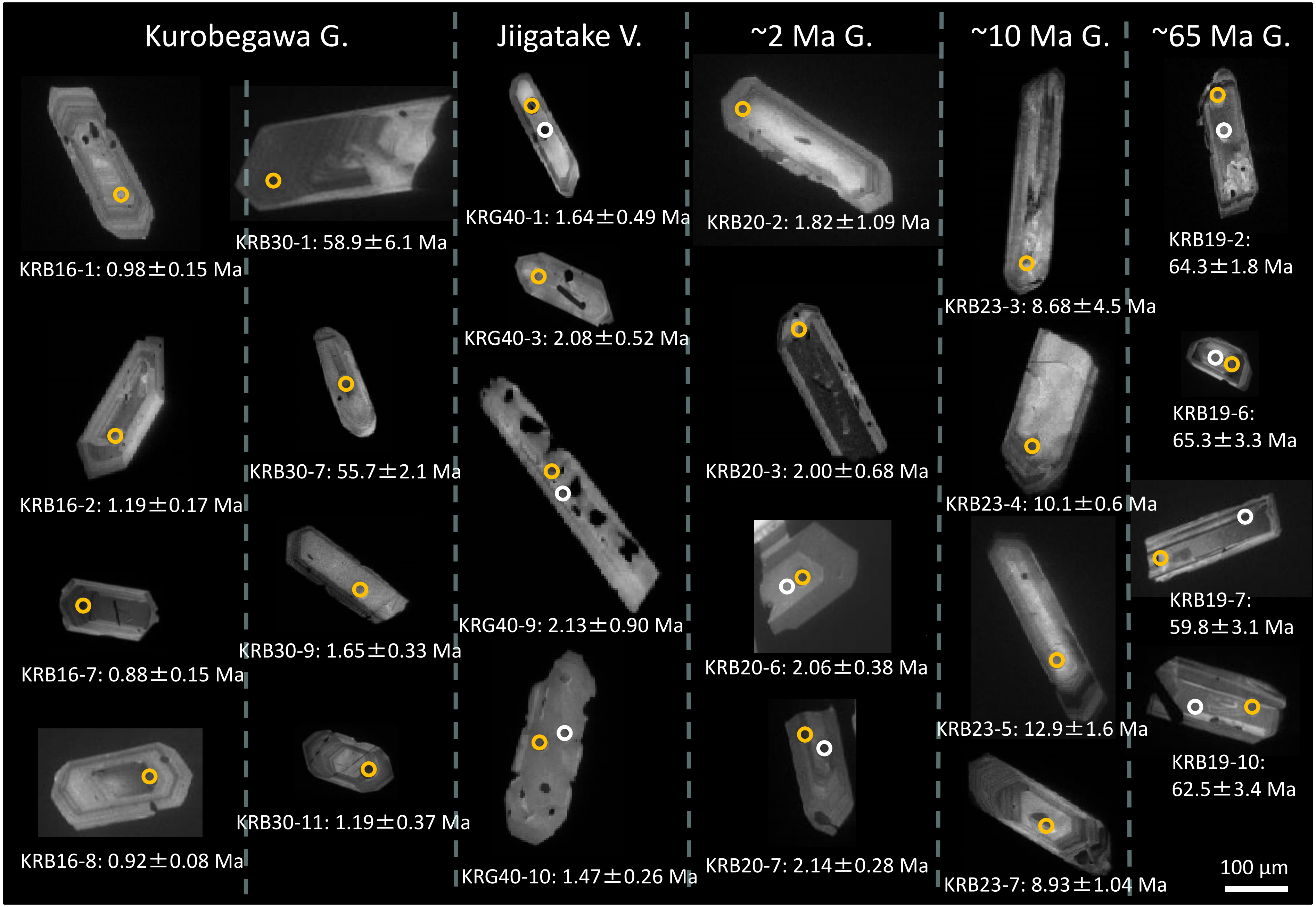

Supplementary Fig. S2. Zircon cathodoluminescence (CL) images with U-Pb ages (2σ error) for representative samples. Orange and white circles denote U-Pb and REE measurement positions, respectively. CL images were obtained using a Hitachi TM4000Plus electron microscope.

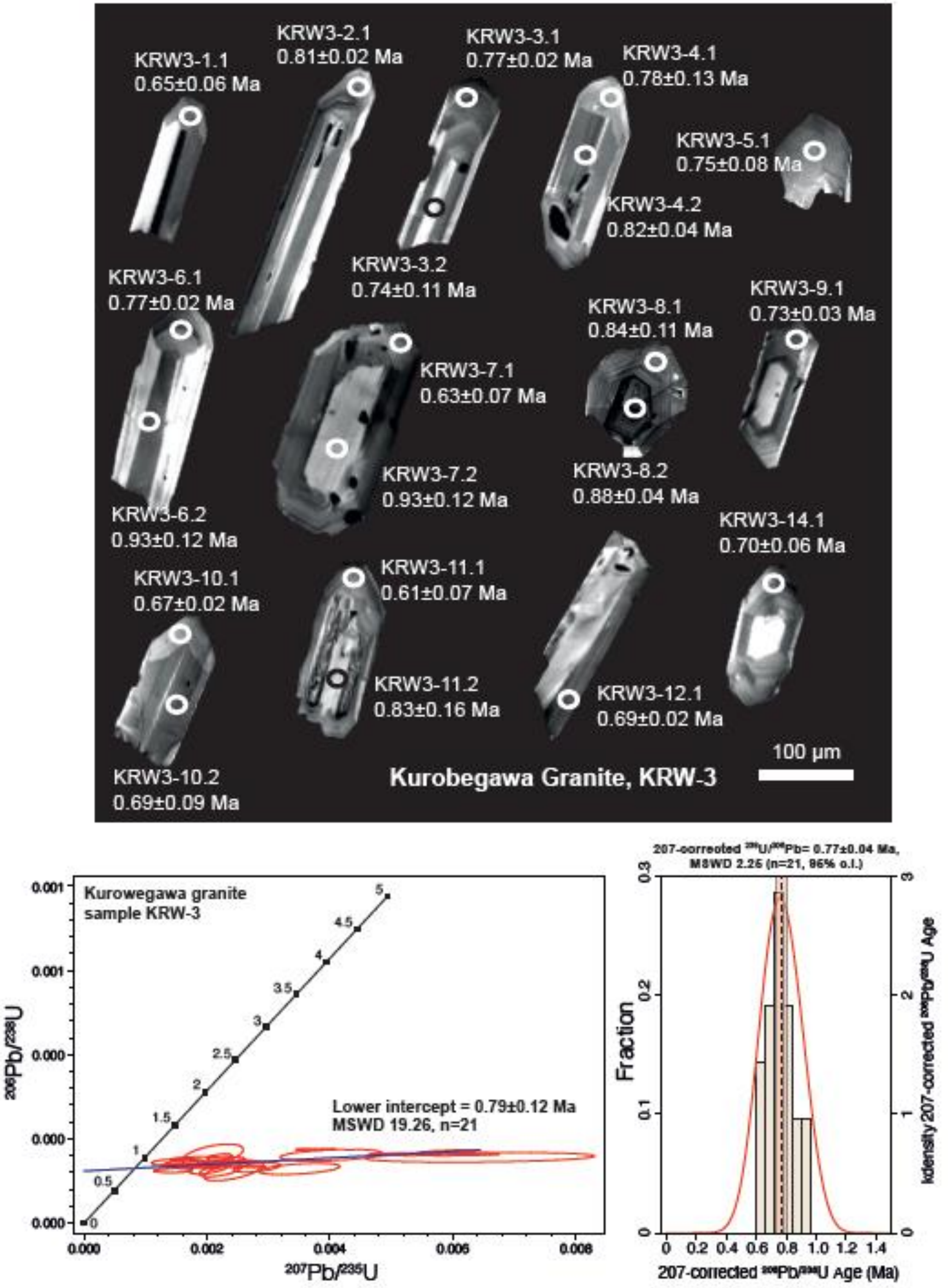

Supplementary Fig. S3. Zircon cathodoluminescence images for KRW3 sample and Wheterill concordia diagram showing SHRIMP results and age estimation after 207-Pb correction.

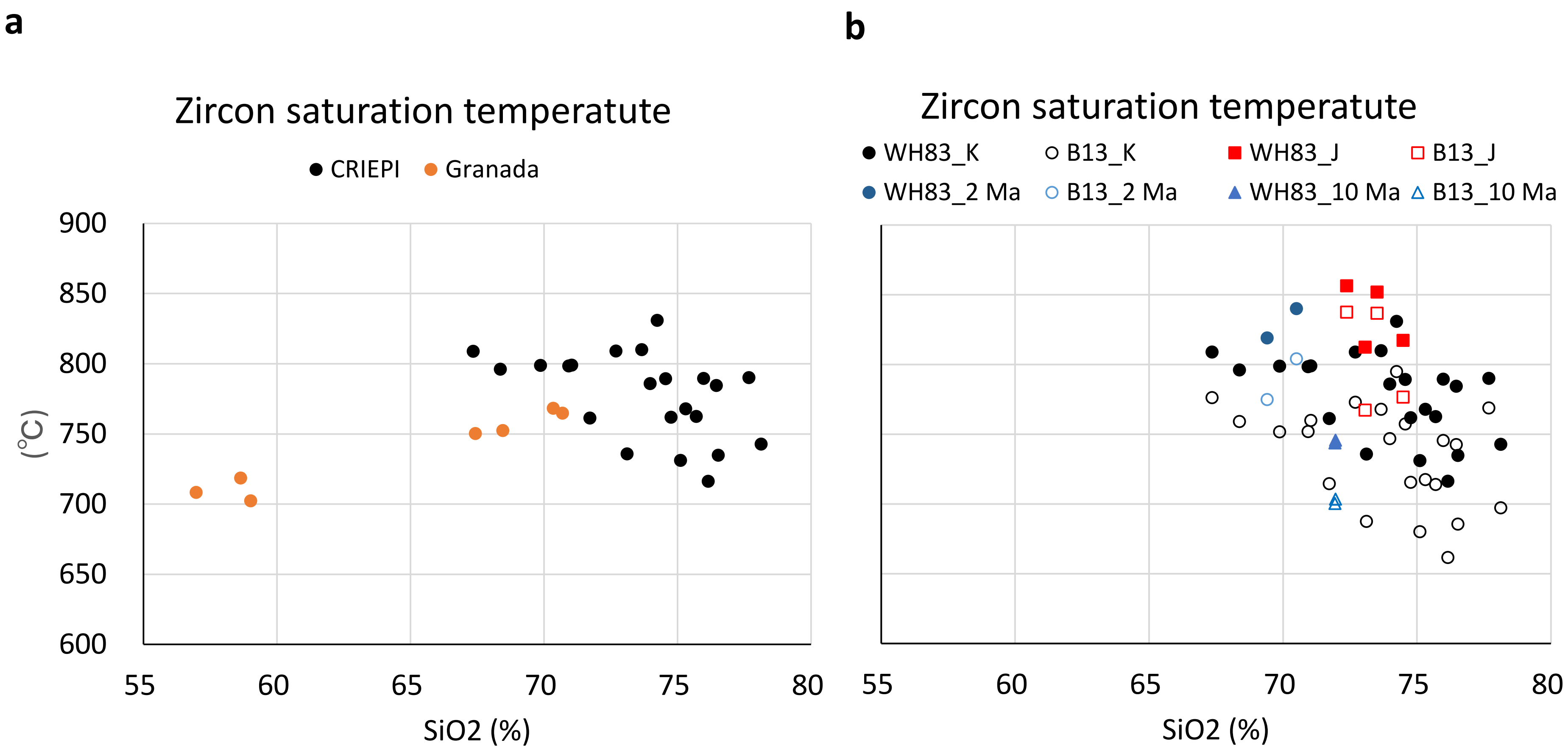

Supplementary Fig. S4. a) Zircon saturation temperature for the Kurobegawa Granite calculated following Watson and Harrison[26]. Black and orange dots represent data obtained from different institutions (CRIEPI and Granada Univ., respectively). b) Zircon saturation temperature calculated following Watson and Harrison[26] and Boehnke et al.[73] using data obtained at CRIEPI. In the legend, WH83: Watson and Harrison[26], B13: Boehnke et al.[73], K: Kurobegawa Granite, J: Jiigatake Volcanics, 2 Ma: 2 Ma granite, 10 Ma: 10 Ma granite.

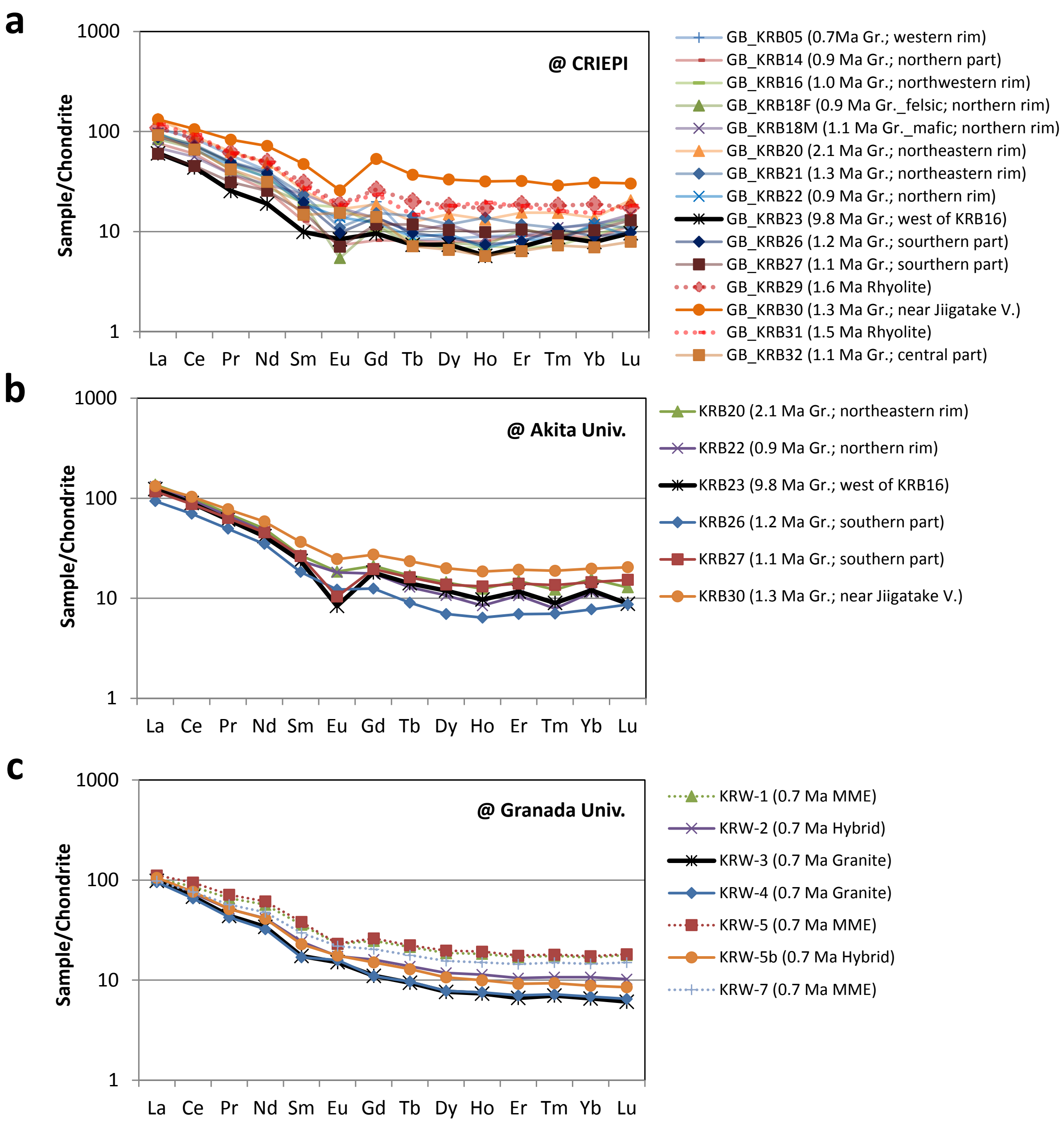

Supplementary Fig. S5. Chondrite normalized REE patterns for whole rock analyzed at CRIEPI (a), Akita Univ. (b), and Granada Univ. (c). In a, dotted lines are for the Jiigatake Volcanics.

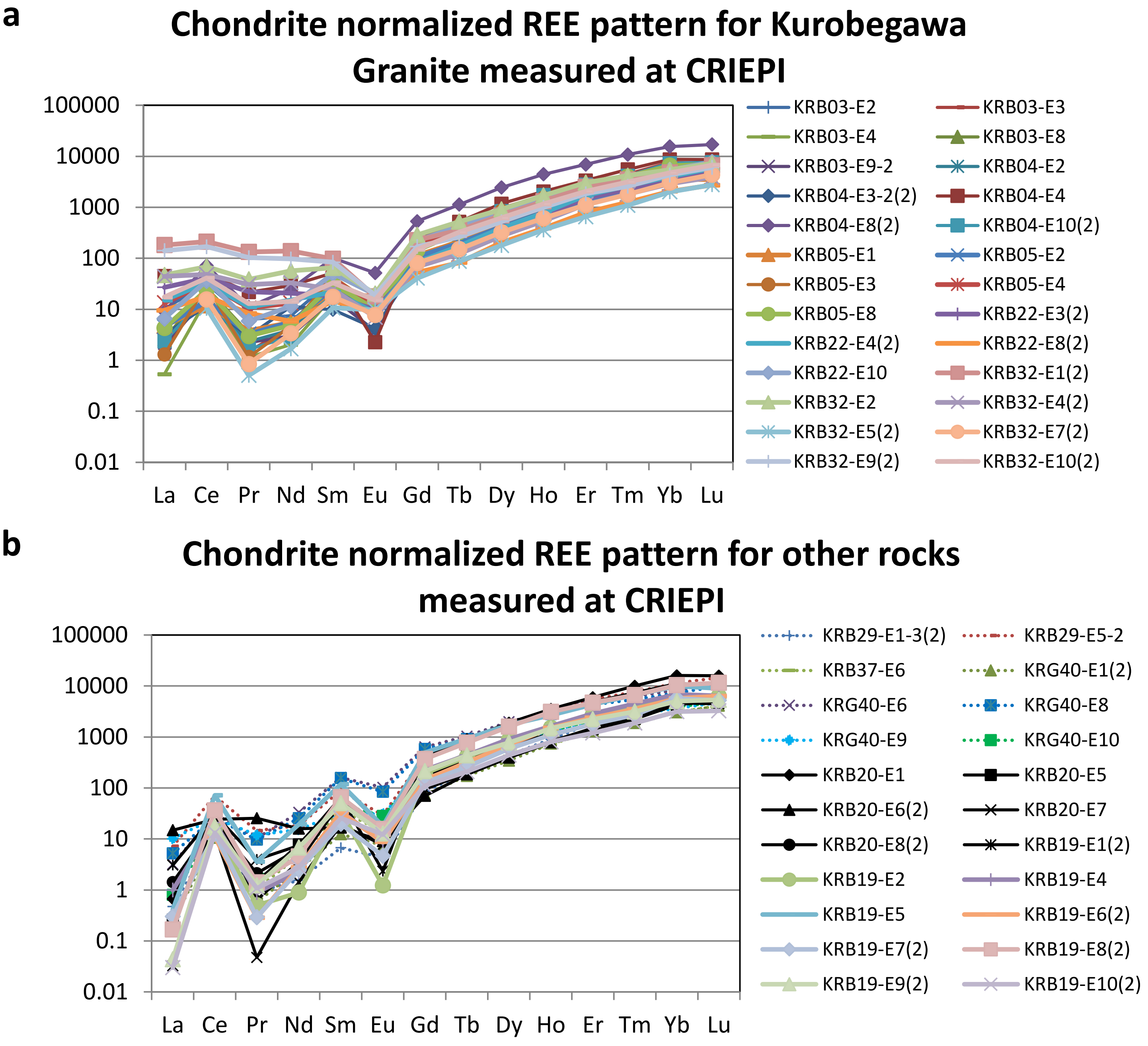

Supplementary Fig. S6. Chondrite normalized REE patterns for zircon. In b, black lines are for 2 Ma granite, dotted lines are for Jiigatake Volcanics, and the others are for 65 Ma granite.

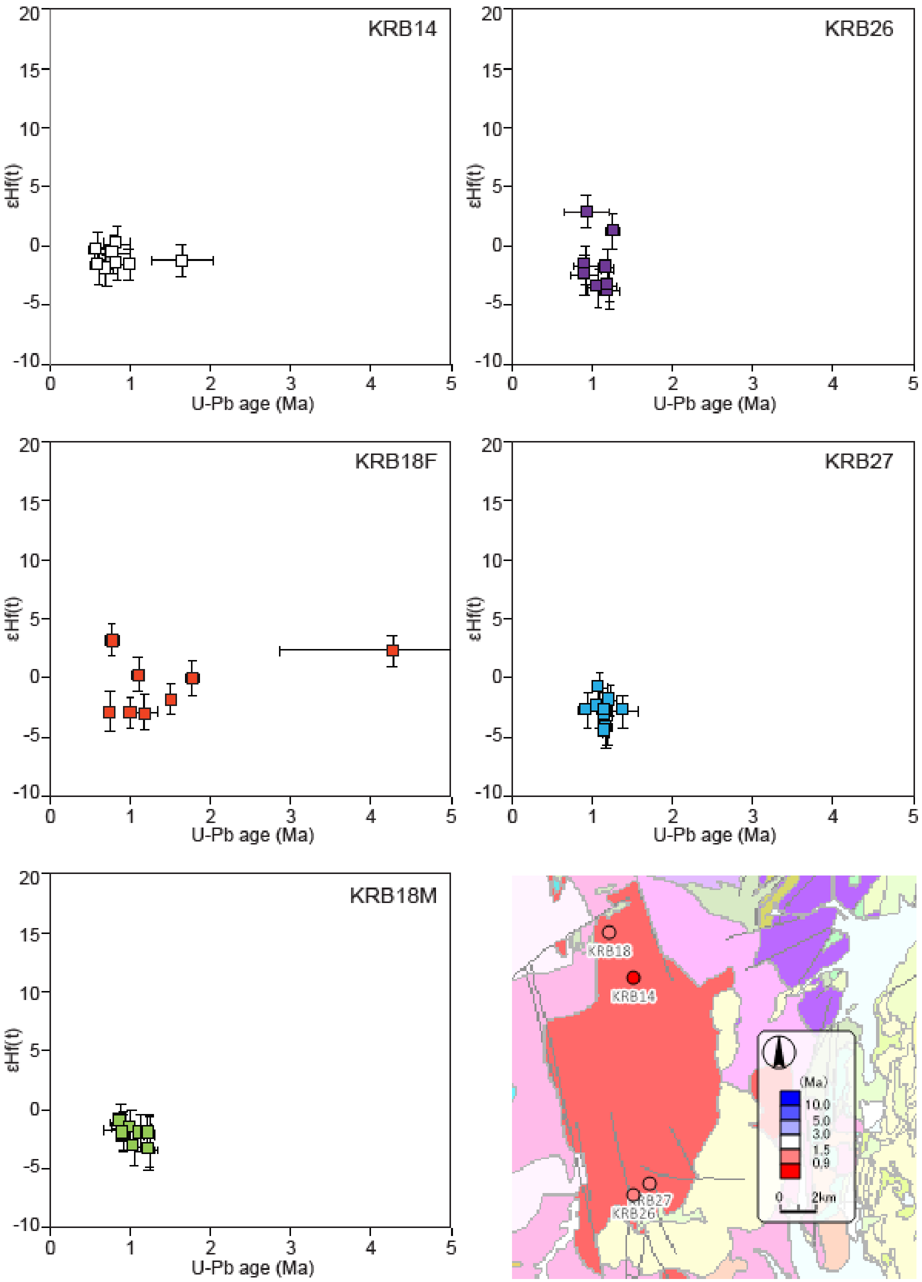

Supplementary Fig. S7. Zircon Hf isotope compositions (shown at 1σ) plotted against U-Pb age. εHf(t) values are shown as initial ratios at time of crystallization. KRB18F and KRB18M are felsic and mafic part of KRB18, respectively. Geological map is modified from Seamless digital geological map of Japan 1: 200,000 (Geological Survey of Japan, AIST) using MANDARA10 (version 10.0.1.5) (https://ktgis.net/mandara/).

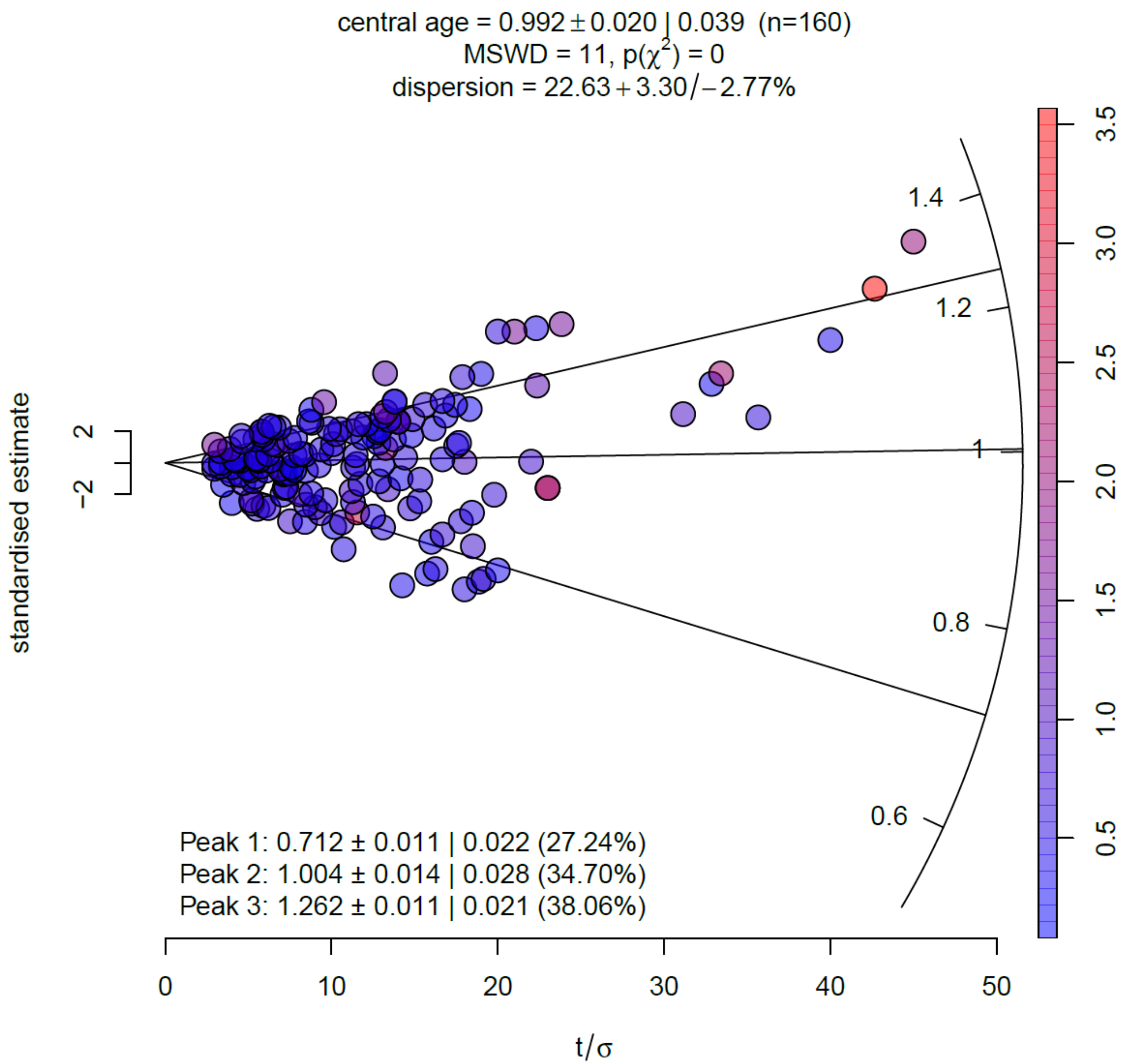

Supplementary Fig. S8. Radial plot of the U-Pb ages for the Kurobegawa Granite. Ages <1.55 Ma are plotted. The colored vertical scale shows Th/U ratio. The plot was drawn using IsoplotR[36].

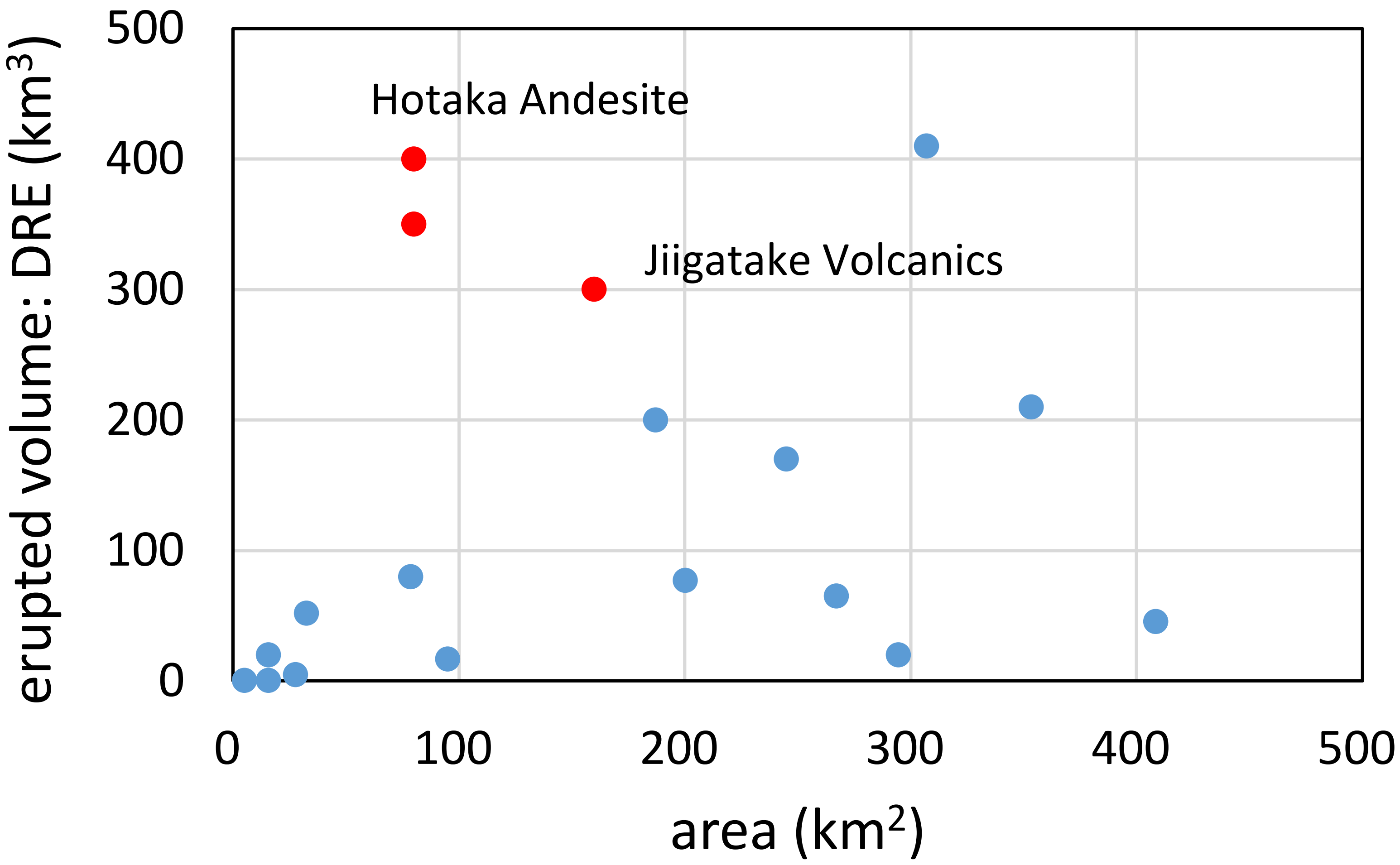

Supplementary Fig. S9. Caldera size and erupted volume (dense rock equivalent: DRE) for Quaternary volcanos in Japan. Data are from Supplementary Table S15.
